# Thymulin restrains age-associated myeloid inflammation and enhances cancer immunotherapy

**DOI:** 10.1038/s41467-026-75383-0

**Published:** 2026-07-21

**Authors:** Hisashi Kanemaru, Steven Luong, Yuta Yamamoto, Yukari Mizukami, Fumito Ito

**Affiliations:** 1 Department of Surgery, Keck School of Medicine of the University of Southern California, Los Angeles, CA USA; 2 Department of Immunology and Immune Therapeutics, Keck School of Medicine of the University of Southern California, Los Angeles, CA USA

**Keywords:** Tumour immunology, Ageing

## Abstract

Chronic inflammation increases with age and contributes to cancer progression and therapeutic resistance, yet the mechanisms underlying this process remain incompletely understood. Here, we identify an increased frequency of pro-inflammatory myeloid cells in aged mice and humans, characterized by elevated production of IL-1α, IL-1β, IL-6, and TNF-α. These cells are enriched in the breast tumor microenvironment and are associated with accelerated tumor progression. Using heterochronic parabiosis and bone marrow chimeras, we show that age-associated myeloid cell inflammatory activation is suppressed by non-bone marrow-derived circulating factors present in young hosts. Integrative analyses identify thymulin, a thymus-derived peptide that declines with age, as a mediator that suppresses pro-inflammatory cytokine production by inhibiting NF-κB signaling. Furthermore, thymulin enhances antitumor T-cell immunity, improves tumor control and survival, and sensitizes tumors to anti-PD-L1 therapy in an age-dependent manner. Together, these findings uncover a thymus–myeloid cell regulatory axis linking aging, inflammation, and cancer immunity, and suggest thymulin as a potential strategy to improve cancer immunotherapy in older individuals.

## Abstract (web summary)

Aging-derived chronic inflammation may drive cancer progression via systemic tumor-associated myeloid cell activation. This study identifies young mice thymulin as suppressing chronic pro-tumorigenic inflammatory features in aged mice.

## Introduction

Aging is one of the most significant risk factors for cancer^1^. The increasing incidence of malignancies in older populations is paralleled by a progressive decline in immune function^2–5^. This decline is characterized by reduced production of naive lymphocytes, accumulation of memory and terminally differentiated B and T cells, and diminished diversity of antibody and T-cell repertoires^2–5^. Another prominent age-related immune remodeling is “inflammaging,” a state of chronic, systemic, sterile, low-level inflammation that occurs with increasing age^6,7^. This concept has emerged as a key contributor not only to cancer but also to a broad spectrum of age-associated diseases, including metabolic disorders, neurodegeneration, chronic kidney disease, and cardiovascular disease. It is characterized by persistent immune activation and elevated circulating levels of pro-inflammatory cytokines, including IL-1α, IL-1β, IL-6, and TNF-α^6,7^. These cytokines are also elevated in solid malignancies such as breast cancer, where they are associated with tumor progression, metastasis, and therapeutic resistance^8,9^. Despite its clinical relevance, the mechanisms driving this age-associated pro-inflammatory state remain incompletely understood.

Accumulating evidence indicates that inhibition of pro-inflammatory cytokine signaling can enhance antitumor efficacy, particularly in combination with immune checkpoint inhibitors (ICIs). Aging is associated with increased systemic IL-6 levels^10^, which correlate with poor response to ICI therapy^11^. Blockade of the IL-6/IL-6 receptor axis mitigates age-associated T-cell dysfunction, promotes Th1 polarization, enhances CD8^+^ T-cell effector differentiation, and synergizes with anti-PD-1 therapy^12–15^. Similarly, increased frequencies of IL-1β^+^ myeloid cells within tumors are associated with resistance to anti-PD-1 therapy, whereas IL-1β blockade reduces myeloid-mediated immunosuppression, delays tumor growth, and enhances responses to checkpoint blockade^8,16^. Enrichment of IL-1α^+^ monocytes and macrophages correlates with aging, poorer survival, and recurrence in lung cancer patients, and blockade of IL-1 receptor type 1 (IL-1R1) improves anti-PD-1 efficacy in preclinical models^17^. TNF-α blockade likewise reduces myeloid-driven immunosuppression^18^, and overcomes resistance to PD-1/PD-L1 blockade in preclinical models of melanoma^19^.

Collectively, these findings suggest that dysregulated inflammation is a major barrier to effective immunotherapy in older individuals and that its therapeutic modulation may enhance clinical outcomes. While complete reversal of age-associated immune dysfunction remains uncertain, studies using heterochronic parabiosis—where young and aged animals share a circulatory system—demonstrate that exposure to a youthful systemic environment reduces inflammatory markers in aged parabionts^20^. Therefore, inflammaging in elderly patients may be attenuated by young blood-derived circulating factors, thereby potentially improving tumor control and enhancing immunotherapy.

In this study, we aim to identify circulating factors derived from young organisms that can attenuate inflammaging and cancer progression, using preclinical models of breast cancer. To this end, we first define the cellular sources of key pro-inflammatory cytokines (IL-1α, IL-1β, IL-6, and TNF-α) in aged mice and humans. We then employ heterochronic parabiosis to test whether systemic factors from young mice suppress the age-associated pro-inflammatory state. To further delineate the origin of these factors, we utilize heterochronic bone marrow chimeras to distinguish between hematopoietic and non-hematopoietic sources. Findings from these analyses and Ingenuity Pathway Analysis implicate the thymus as a regulator of age-associated myeloid inflammation and identify thymulin as a circulating factor that suppresses myeloid cell-derived inflammatory cytokine production, delays tumor growth, and enhances the efficacy of immune checkpoint blockade in aged mice.

## Results

### Pro-inflammatory cytokine-producing myeloid cells are increased in aged mice and are associated with accelerated tumor progression

**Fig. 1:**
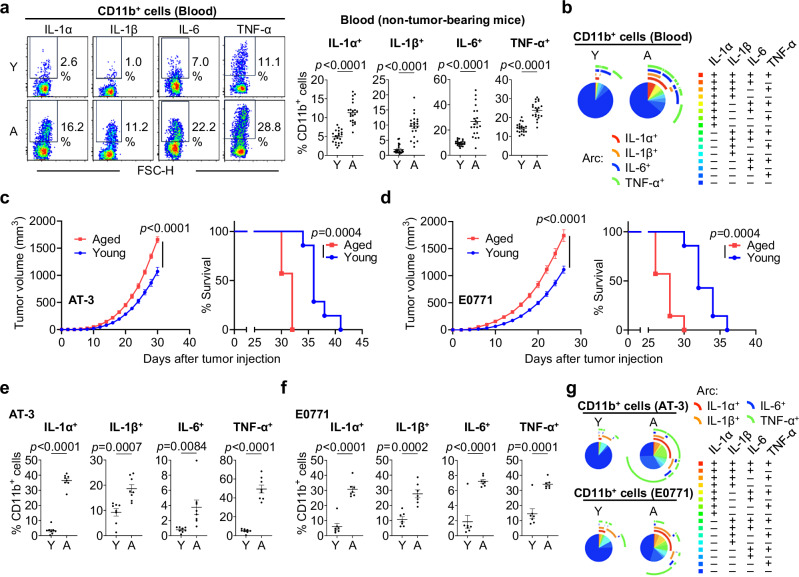
Pro-inflammatory cytokine-producing myeloid cells are increased in aged mice and are associated with accelerated tumor progression. **a**, **b** Representative flow cytometry plots (**a**, left) and frequency (**a**, right) of cytokine (IL-1α, IL-1β, IL-6, and TNF-α)-producing cells within CD11b^+^ blood cells from non-tumor-bearing young (Y) and aged (A) C57BL/6 female mice (*n* = 21 per group), with representative Boolean analysis (**b**). **c**, **d** Tumor growth curves (mean, left) and survival curves (right) of female mice bearing AT-3 (**c**, *n* = 7 per group) and E0771 (**d**, *n* = 7 per group) tumors. *P*-values were determined by two-way ANOVA with Bonferroni’s multiple comparisons test (tumor growth) and log-rank test (survival). **e**–**g** Frequency of cytokine-producing cells within AT-3 (**e**, *n* = 8 per group) and E0771 (**f**, *n* = 7 per group) tumor-infiltrating CD11b^+^ cells from young and aged mice, with representative Boolean analysis (**g**). Statistical significance was assessed using two-tailed *t*-tests (**a**, **e**, **f**). Mean ± SEM. *n* values represent independent mice.

To identify the cellular source of pro-inflammatory cytokines contributing to inflammaging, we examined the expression of IL-1α, IL-1β, IL-6 and TNF-α in hematopoietic cells from non-tumor-bearing young (8–12 weeks) and aged (65–75 weeks) C57BL/6 female mice by flow cytometry (Supplementary Fig. 1). We observed a marked increase in myeloid cell populations producing pro-inflammatory cytokines in aged mice, including Ly6C^+^ Ly6G^−^ monocytes, Ly6C^+^ Ly6G^+^ granulocytes, Ly6C^−^ Ly6G^−^ F4/80^+^ I-A/I-E^+^ macrophages and Ly6C^−^ Ly6G^−^ F4/80^−^ I-A/I-E^+^ CD11c^+^ dendritic cells (DCs) among circulating CD45^+^ cells, compared with young mice (Fig. 1a, b and Supplementary Fig. 2). A similar age-associated increase in circulating cytokine-producing myeloid cells was observed in C57BL/6 male mice (Supplementary Fig. 3a, b).

To assess the functional relevance of these changes, we utilized two syngeneic orthotopic breast cancer mouse models, AT-3 and E0771. Aged female mice exhibited accelerated tumor progression and reduced survival compared with young mice (Fig. 1c, d). Consistent with this, enhanced tumor progression was also observed in aged male mice bearing B16-F10 melanomas (Supplementary Fig. 3c). A modest increase in the frequency of circulating pro-inflammatory myeloid cells was observed in tumor-bearing mice compared with non-tumor-bearing controls in both young and aged cohorts (Supplementary Fig. 4a, b), indicating that tumor presence contributes to systemic inflammation. However, this effect was less pronounced than the age-associated increase observed between young and aged mice (Supplementary Fig. 4c, d). Although the overall frequency of intratumoral CD45⁺ cells was comparable between young and aged tumor-bearing mice (Supplementary Fig. 5a), CD11b^+^ myeloid cells producing pro-inflammatory IL-1α, IL-1β, IL-6, and/or TNF-α were substantially increased in tumors from aged mice (Fig. 1e–g). Within the CD11b^+^ compartment, monocytes and macrophages represented the predominant cytokine-producing subsets in aged mice (Supplementary Fig. 5b–d).

### Age-associated pro-inflammatory myeloid cell alterations in human peripheral blood and breast tumors

**Fig. 2:**
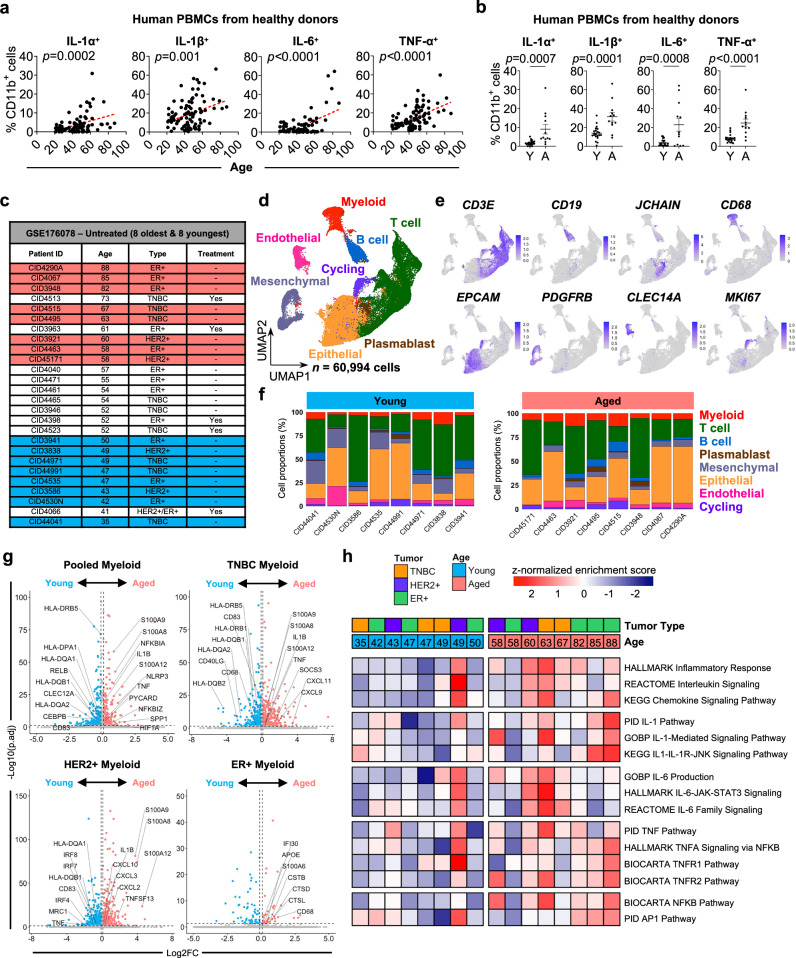
Age-associated pro-inflammatory myeloid cell alterations in human peripheral blood and breast tumors. **a**, **b** Flow cytometry analysis of peripheral blood mononuclear cells (PBMCs) from healthy donors. **a** Correlation between age and the frequency of cytokine (IL-1α, IL-1β, IL-6, and TNF-α)-producing cells within CD33^+^ CD11b^+^ PBMCs (*n* = 90). **b** Frequency of cytokine-positive cells among CD33⁺ CD11b⁺ PBMCs in young (21–33 years; Y, *n* = 21) and aged (60–87 years; A, *n* = 12) donors. **c** Clinical characteristics of 26 primary breast tumors (GSE176078), including patient ID, age, tumor type, and treatment status. Among treatment-naïve patients, the eight youngest (blue) and eight oldest (red) were selected for downstream analyses. **d** UMAP visualization of cell clusters from 16 tumor samples. **e** Expression plots of canonical lineage markers to define major cell populations. **f** Relative proportions of major cell types in tumors from young and aged patients. **g** Volcano plots of differentially expressed genes in intratumoral myeloid cells comparing young and aged patients, including analyses across all myeloid cells and stratified by subtype (TNBC, HER2⁺, ER⁺). Each red and blue dot denotes an individual gene with Benjamini–Hochberg-adjusted *p*-value < 0.05 and log fold change > 0.10. **h** Heatmap of z-score–normalized pathway enrichment in intratumoral myeloid cells across individual patients. Statistical significance was assessed using two-tailed Pearson correlation (**a**) or two-tailed *t*-tests (**b**). Mean ± SEM. *n* values represent independent healthy donors (**a**, **b**). TNBC: triple-negative breast cancer, ER⁺: estrogen receptor positive, HER2⁺: human epidermal growth factor receptor 2–positive.

We next evaluated whether age-associated expansion of pro-inflammatory myeloid cells is conserved in humans. Using peripheral blood mononuclear cells (PBMCs) from healthy donors, we assessed the frequency of CD33⁺CD11b⁺ myeloid cells producing IL-1α, IL-1β, IL-6, and/or TNF-α (Supplementary Fig. 6). The frequency of cytokine-producing myeloid cells positively correlated with age (Fig. 2a) and was significantly increased in older individuals (60–87 years) compared with younger individuals (21–33 years) (Fig. 2b).

To determine whether a similar age-associated pro-inflammatory myeloid profile is present within the tumor microenvironment, we analyzed a publicly available single-cell RNA sequencing dataset of human breast cancer^21^ comprising 26 primary breast tumors, including 10 triple-negative breast cancer (TNBC), 11 estrogen receptor positive (ER+), and 5 HER2+ tumors, spanning an age range of 35–88 years. Patients were stratified into young (35–50 years; mean 45.3 years; *n* = 8) and aged (58–88 years; mean 70.1 years; *n* = 8) groups with exclusion of individuals who received neoadjuvant therapy to avoid potential treatment-induced alterations in the tumor microenvironment (Fig. 2c). After quality control and doublet removal, we obtained 60,994 high-quality cells and identified major cell populations using canonical lineage markers, including *EPCAM*^+^ epithelial cells, *CLEC14A*^+^ endothelial cells, *PDGFRB*^+^ mesenchymal cells, *CD19*^+^ B cells, *JCHAIN*^+^ plasmablasts, *MKI67*^+^ cycling cells, *CD3E*^+^ T cells, and *CD68*^+^ myeloid cells (Fig. 2d, e). Comparative analysis revealed a relative increase in the frequency of myeloid cells in tumors from aged patients compared with younger patients (Fig. 2f).

To further characterize age-associated differences, we performed differential gene expression analysis on pooled intratumoral myeloid cells (*n* = 4035 cells from 16 patients). Myeloid cells from younger patients were enriched for genes associated with antigen presentation and maturation, including *HLA-DRB5*, *HLA-DPA1*, *HLA-DQA1*, *HLA-DQA2*, *HLA-DQB1*, and *CD83*. In contrast, myeloid cells from aged patients exhibited increased expression of pro-inflammatory cytokines (*IL1B* and *TNF)*, NF-κB pathway regulators (*NFKBIA*, *NFKBIZ*), damage-associated molecular pattern (DAMP) molecules (*S100A8*, *S100A9*, *S100A12*), and inflammasome components (*NLRP3*, *PYCARD*) (Fig. 2g and Supplementary Dataset 1), consistent with inflammatory myeloid states implicated in aging and age-related pathologies^22–25^. Stratification by breast cancer subtype—TNBC (young: 893 cells; aged: 1066 cells), ER⁺ (young: 295 cells; aged: 751 cells), and HER2⁺ (young: 491 cells; aged: 539 cells)—suggested a similar pro-inflammatory transcriptional profile in myeloid cells from aged patients across subtypes. Consistent with these findings, pathway analysis demonstrated enrichment of inflammatory signaling pathways, including IL-1, IL-6, and TNF signaling, as well as downstream NF-κB and AP-1 activation (Fig. 2h and Supplementary Dataset 2). Collectively, these results demonstrate an age-dependent expansion and activation of pro-inflammatory myeloid cells in both the peripheral circulation and tumor microenvironment in humans.

### Exposure to a youthful systemic environment attenuates myeloid cell inflammatory activation and tumor progression in aged mice

**Fig. 3:**
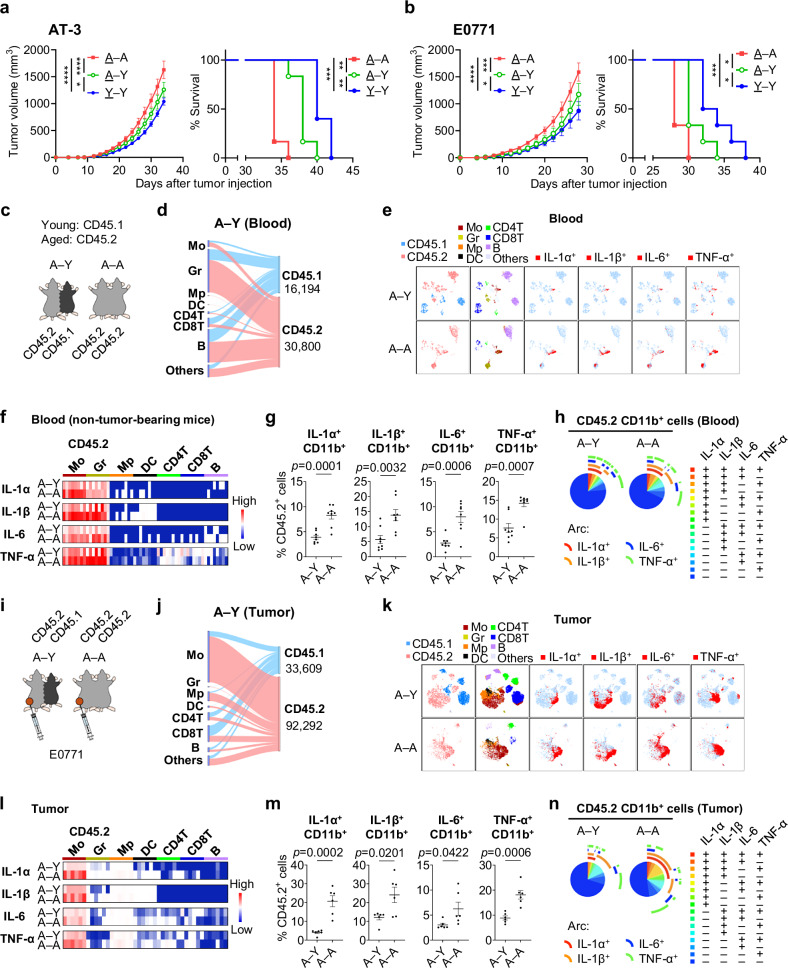
Exposure to a youthful systemic environment attenuates myeloid cell inflammatory activation and tumor progression in aged mice. **a**, **b** Tumor growth curves (mean, left) and survival curves (right) of AT-3 (**a**, A–A and A–Y, *n* = 6; Y–Y, *n* = 5) and E0771 (**b**, *n* = 6 per group) tumor-bearing parabiotic mice. Y: young, A: aged. Underlined labels indicate tumor-bearing parabionts. *p < 0.05; **p < 0.01; ***p < 0.001; ****p < 0.0001 by two-way ANOVA with Bonferroni’s multiple comparisons test (tumor growth) and log-rank test (survival). Exact *p*-values are provided in the Source Data. **c** Experimental schematic of congenic parabiosis using CD45.1 (young) and CD45.2 (aged) mice. **d**–**h** Flow cytometry analysis of circulating CD45.1^+^ young- and CD45.2^+^ aged-derived cells 2 weeks after establishment of parabiosis (*n* = 8 pairs). **d** Sankey diagram showing the distribution of blood leukocyte subsets across CD45.1 and CD45.2 lineages in heterochronic (A–Y) pairs. Data represent pooled cells from 8 parabiotic pairs. **e** Representative UMAP image of CD45.1⁺ and CD45.2⁺ blood cells. **f**–**h** Frequency of cytokine-producing cells within CD45.2^+^ blood cells, shown as a heatmap (**f**) and quantified in data panels (**g**), with representative Boolean analysis (**h**). **i** Experimental schematic of tumor-bearing parabiosis. **j**–**n** Flow cytometry analysis of CD45.1^+^ young- and CD45.2^+^ aged-derived cells in E0771 tumors in aged parabiont (*n* = 6 pairs). **j** Sankey diagram showing the distribution of tumor-infiltrating immune cell populations across CD45.1 and CD45.2 lineages in A–Y pairs. **k** Representative UMAP image of tumor-infiltrating CD45.1⁺ and CD45.2⁺ cells. **l**–**n** Frequency of cytokine-producing cells within tumor-infiltrating CD45.2^+^ cells, shown as a heatmap (**l**) and quantified in data panels (**m**), with representative Boolean analysis (**n**). Statistical significance was assessed using two-tailed *t*-test (**g**, **m**). Mean ± SEM. Mo: monocytes, Gr: granulocytes, Mp: macrophages, DC: dendritic cells, CD4T: CD4⁺ T cells, CD8T: CD8⁺ T cells, B: B cells. *n* values represent independent parabiotic pairs.

Previous studies have demonstrated that exposure to a young systemic milieu can reverse age-associated phenotypes, including chronic inflammation^26^. To determine whether the dysregulated pro-inflammatory state in aged mice can be modulated by circulating factors from young mice, we employed parabiosis, in which two animals are surgically joined to share a common circulatory system^20^. We established heterochronic parabiotic pairs between aged and young mice (A–Y), as well as isochronic controls consisting of young-young (Y–Y) and aged-aged (A–A) pairs (Supplementary Fig. 7a). In non-tumor-bearing mice, the frequency of circulating cytokine-producing myeloid cells in A–Y pairs was reduced to levels comparable to those observed in Y–Y pairs (Supplementary Fig. 7b, c). In tumor-bearing mice, A–Y pairing similarly resulted in a marked reduction in circulating pro-inflammatory myeloid cells (Supplementary Fig. 7d–f), accompanied by delayed progression of tumors in aged parabionts and improved survival compared with A–A pairs (Fig. 3a, b). Conversely, increased progression of tumors in young parabionts and worse survival were observed in A–Y pairs compared with Y–Y pairs (Supplementary Fig. 7g, h).

To assess whether these effects reflect changes intrinsic to aged myeloid cells, we utilized a CD45.1/CD45.2 congenic system to distinguish young (CD45.1)- and aged (CD45.2)-derived hematopoietic cells (Fig. 3c). Analysis of blood CD45.1/CD45.2 chimerism after 2 weeks of parabiosis demonstrated approximately 40% exchange of circulating leukocytes between parabionts (Fig. 3d), consistent with a previous report^27^. Notably, expression of IL-1α, IL-1β, IL-6, and/or TNF-α in aged-derived CD45.2 myeloid cells was substantially reduced in A–Y pairs compared with A–A controls (Fig. 3e–h). We next assessed intratumoral myeloid cells in congenic parabiotic pairs (Fig. 3i, j). Consistent with peripheral findings, aged-derived (CD45.2⁺) intratumoral myeloid cells from A–Y pairs exhibited reduced expression of pro-inflammatory cytokines relative to those from A–A pairs (Fig. 3k–n). Together, these results demonstrate that exposure to a youthful systemic environment suppresses myeloid cell inflammatory activation and reduces tumor progression in aged mice.

### Non-bone marrow-derived circulating factors in young hosts regulate pro-inflammatory cytokine production in aged myeloid cells

**Fig. 4:**
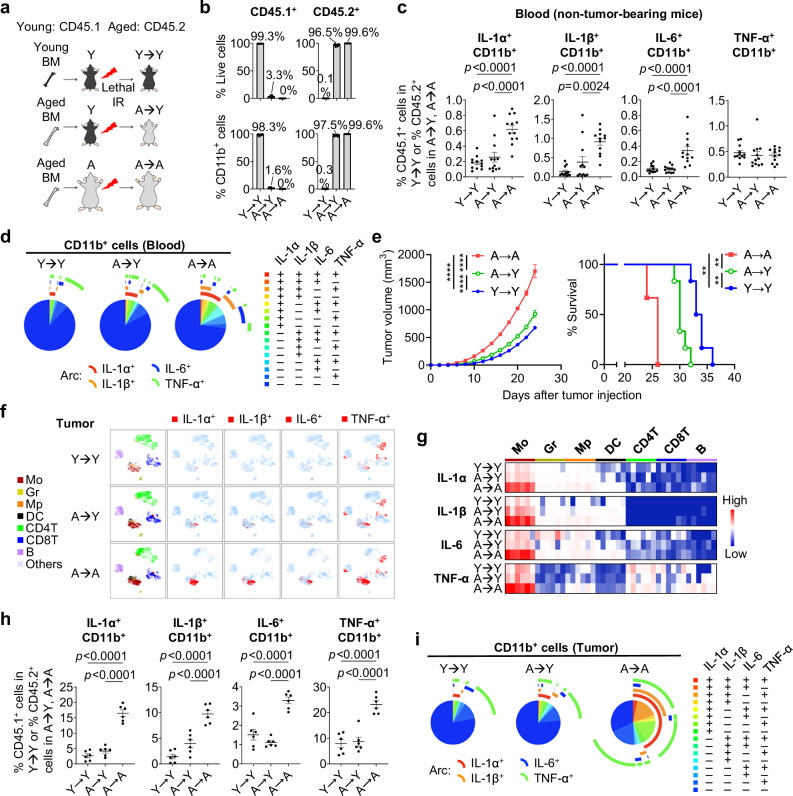
Non–bone marrow-derived circulating factors in young hosts regulate pro-inflammatory cytokine production in aged myeloid cells. **a**–**d** Young (Y; CD45.1) or aged (A; CD45.2) recipient mice were irradiated (IR) and reconstituted with bone marrow from young or aged donors to generate Y → Y, A → Y, and A → A chimeras. **a** Experimental schematic of bone marrow chimera generation. **b** Donor chimerism showing the proportions of CD45.1⁺ and CD45.2⁺ cells among total live cells and within the CD11b⁺ myeloid compartment (*n* = 12 per group). **c**, **d** Frequency of cytokine-producing cells within CD45.1⁺ (Y → Y) or CD45.2⁺ (A → Y, A → A) blood cells, shown as quantified data (**c**, *n* = 12 per group), with representative Boolean analysis (**d**). **e** Tumor growth curves (mean, left) and survival curves (right) of E0771 tumor-bearing chimeric mice (*n* = 6 per group). **p < 0.01; ****p < 0.0001 by two-way ANOVA with Bonferroni’s multiple comparisons test (tumor growth) and log-rank test (survival). Exact *p*-values are provided in the Source Data. **f**–**i** Flow cytometry analysis of CD45.1^+^ young and CD45.2^+^ aged bone marrow-derived cells in E0771 tumors. **f** Representative UMAP image of CD45.1⁺ (Y → Y) or CD45.2⁺ (A → Y, A → A) tumor-infiltrating cells from chimeric mice. **g**–**i** Frequency of cytokine-producing cells within each CD45.1⁺ (Y → Y) or CD45.2⁺ (A → Y, A → A) tumor-infiltrating cells (*n* = 6 per group), shown as a heatmap (**g**) and quantified in data panels (**h**), with representative Boolean analysis (**i**). Statistical significance was assessed using one-way ANOVA with Tukey’s multiple comparisons (**c**, **h**). Mean ± SEM. Mo: monocytes, Gr: granulocytes, Mp: macrophages, DC: dendritic cells, CD4T: CD4⁺ T cells, CD8T: CD8⁺ T cells, B: B cells. *n* values represent independent mice.

We next asked whether circulating factors from young mice that mitigate age-associated myeloid cell inflammatory activation are derived from hematopoietic or non-hematopoietic sources. To address this, we generated bone marrow chimeras comprising young-to-young (Y → Y), aged-to-young (A → Y), and aged-to-aged (A → A) combinations (Fig. 4a) and assessed cytokine production by donor-derived myeloid cells and tumor progression. Engraftment efficiency was comparable across groups, with similar levels of donor chimerism within the CD11b⁺ myeloid compartment (Fig. 4b). In A → Y chimeras, expression of IL-1α, IL-1β, and IL-6 in CD45.2⁺ aged bone marrow-derived circulating CD11b^+^ myeloid cells was markedly reduced compared with A → A controls, reaching levels comparable to those in CD45.1⁺ young bone marrow-derived myeloid cells in Y → Y chimeras (Fig. 4c, d). Consistent with this, A → Y mice exhibited delayed tumor growth and prolonged survival relative to A → A mice (Fig. 4e). In the tumor microenvironment, A → Y chimeras similarly showed a reduced frequency of CD11b⁺ myeloid cells producing pro-inflammatory cytokines compared with A → A controls (Fig. 4f–i). These findings indicate that non-bone marrow-derived circulating factors in young hosts play an important role in suppressing pro-inflammatory cytokine production in aged bone marrow-derived myeloid cells and limiting tumor progression.

To further assess the contribution of the host environment, we generated young-to-aged (Y → A) chimeras (Supplementary Fig. 8a, b). In this setting, both circulating and intratumoral young bone marrow-derived myeloid cells exhibited increased pro-inflammatory cytokine production in Y → A chimeras compared with their counterparts in Y → Y chimeras (Supplementary Fig. 8c–h), indicating that the aged host environment promotes inflammatory activation of myeloid cells irrespective of their origin.

### Host age dictates myeloid cell inflammatory activation in heterochronic mixed bone marrow chimeras

**Fig. 5:**
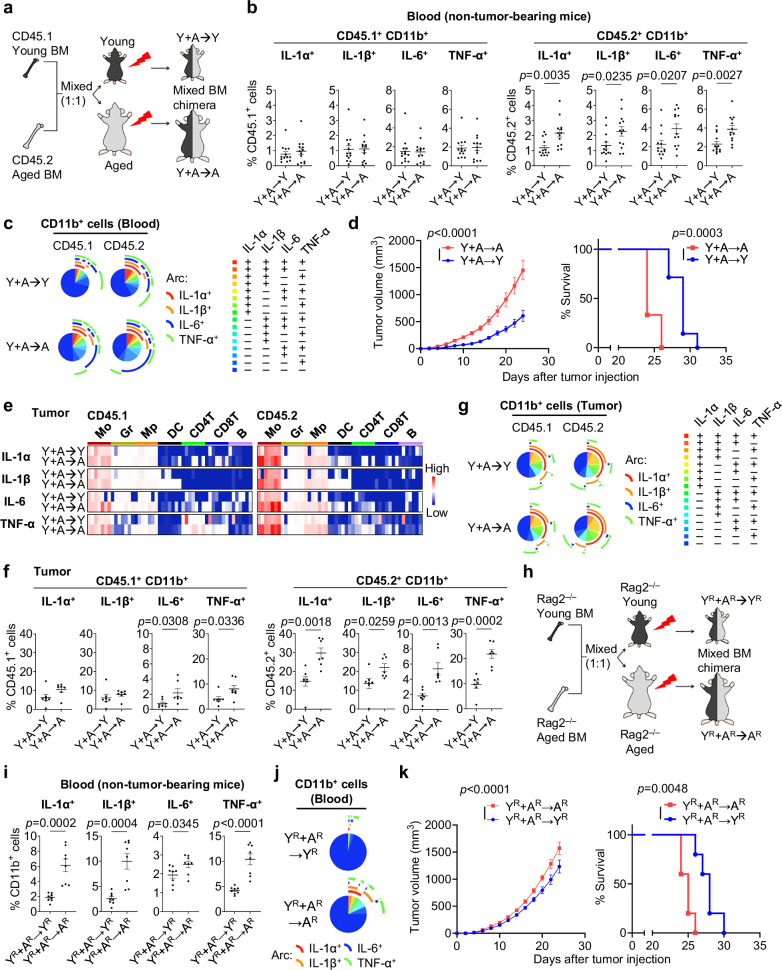
Host age dictates myeloid cell inflammatory activation in heterochronic mixed bone marrow chimeras. **a** Experimental schematic of heterochronic mixed bone marrow chimeras. Irradiated young (Y + A → Y) or aged (Y + A → A) recipient mice were reconstituted with a 1:1 mixture of bone marrow cells from young (CD45.1) and aged (CD45.2) donors. **b**, **c** Frequency of cytokine-producing cells within CD45.1⁺ and CD45.2⁺ blood cells from mixed chimeric mice (*n* = 13 per group), shown as quantified data (**b**), with representative Boolean analysis (**c**). **d** Tumor growth curves (mean, left) and survival curves (right) of E0771 tumor-bearing mixed chimeric mice (Y + A → Y, *n* = 7; Y + A → A, *n* = 6). **e**–**g** Frequency of cytokine-producing cells within CD45.1⁺ and CD45.2⁺ tumor-infiltrating cells (*n* = 7 per group), shown as a heatmap (**e**) and quantified in data panels (**f**), with representative Boolean analysis (**g**). **h** Experimental schematic of Rag2-deficient (Rag2^⁻/⁻^) heterochronic mixed bone marrow chimeras. Irradiated young (Y^R^ + A^R^ → Y^R^) or aged (Y^R^ + A^R^ → A^R^) recipient mice were reconstituted with a 1:1 mixture of bone marrow cells from young and aged Rag2^⁻/⁻^ donors. **i**, **j** Frequency of cytokine-producing cells within CD11b^+^ blood cells from Rag2^⁻/⁻^ mixed chimeric mice (*n* = 8 per group), shown as quantified data (**i**), with representative Boolean analysis (**j**). **k** Tumor growth curves (mean, left) and survival curves (right) of E0771 tumor-bearing Rag2^⁻/⁻^ mixed chimeric mice (*n* = 5 per group). Statistical significance was assessed using two-tailed *t*-test (**b**, **f**, **i**), two-way ANOVA with Bonferroni’s multiple comparisons test (tumor growth), and log-rank test (survival) (**d**, **k**). Mean ± SEM. Mo: monocytes, Gr: granulocytes, Mp: macrophages, DC: dendritic cells, CD4T: CD4⁺ T cells, CD8T: CD8⁺ T cells, B: B cells. *n* values represent independent mice.

Our bone marrow chimera studies suggested that non-bone marrow-derived circulating factors from young hosts suppress age-associated myeloid cell inflammatory activation, while also implicating host age as a key determinant of this phenotype. To directly test this, we generated heterochronic young (CD45.1)/aged (CD45.2) mixed bone marrow chimeras into either young mice (Y + A → Y) or aged mice (Y + A → A) (Fig. 5a) and evaluated bone marrow-derived myeloid cells and tumor growth. Engraftment efficiency was comparable between young and aged donor cells, with similar levels of chimerism within the CD11b⁺ myeloid compartment across groups (Supplementary Fig. 9a). In young hosts (Y + A → Y), expression of IL-1α, IL-1β, IL-6, and/or TNF-α in aged-derived CD45.2 myeloid cells was markedly reduced compared with aged hosts (Y + A → A) (Fig. 5b, c). In contrast, cytokine expression in young-derived (CD45.1⁺) myeloid cells was relatively similar between hosts, indicating that the host environment predominantly governs inflammatory activation of aged myeloid cells.

We next evaluated tumor progression in these chimeras. Tumor growth was significantly delayed and survival improved in Y + A → Y mice compared with Y + A → A mice (Fig. 5d). Consistent with this, the frequency of intratumoral aged-derived CD45.2 myeloid cells producing pro-inflammatory cytokines was substantially reduced in Y + A → Y mice relative to Y + A → A mice (Fig. 5e–g).

We observed differences in immune composition between Y + A → Y and Y + A → A chimeras, with Y + A → Y mice exhibiting increased T-cell abundance, potentially reflecting differences in thymic function (Supplementary Fig. 9b–d). To determine whether the observed effects on tumor progression and myeloid cell activation were independent of adaptive immunity, we generated heterochronic mixed bone marrow chimeras using Rag2-deficient mice as donors and recipients (Fig. 5h). In the absence of T and B cells, the overall frequency of circulating CD11b^+^ myeloid cells was comparable between Y + A → Y and Y + A → A chimeras (Supplementary Fig. 10a, b). However, Y + A → Y mice still exhibited delayed tumor progression, improved survival, and reduced frequencies of circulating cytokine-producing CD11b⁺ myeloid cells compared with Y + A → A mice (Fig. 5i–k and Supplementary Fig. 10c, d). Together, these findings demonstrate that host age dictates the pro-inflammatory state of the myeloid compartment and that non-bone marrow-derived circulating factors from young hosts play an important role in restraining myeloid inflammation and tumor progression.

### Thymulin suppresses pro-inflammatory cytokine production in aged myeloid cells via inhibition of NF-κB signaling

**Fig. 6:**
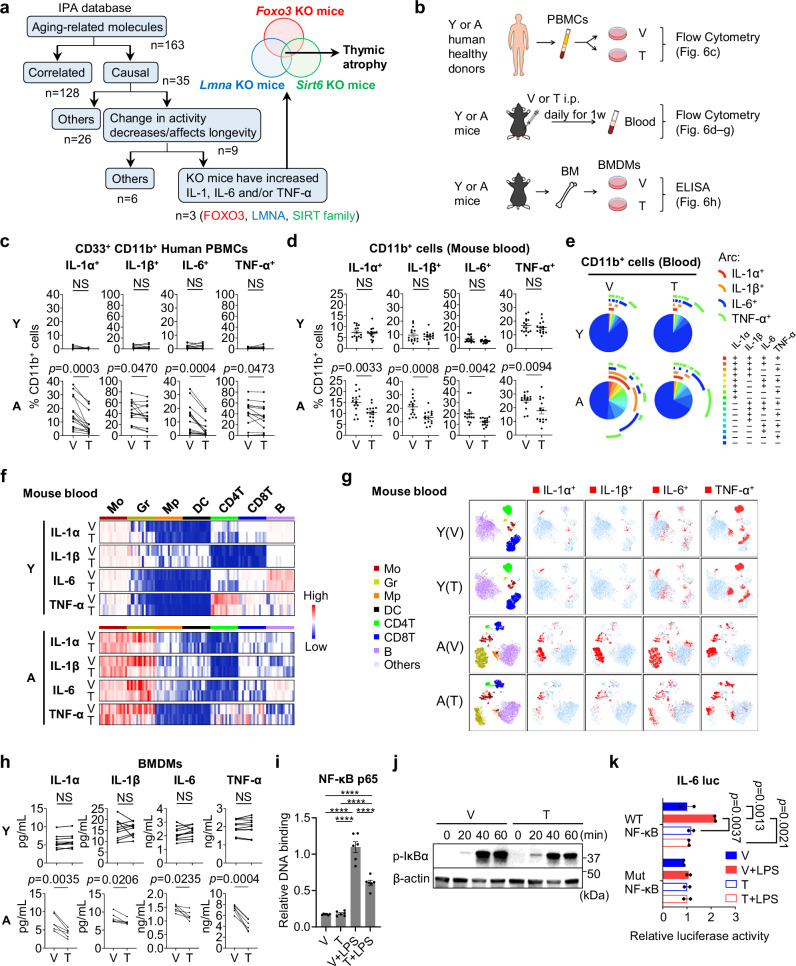
Thymulin suppresses pro-inflammatory cytokine production in aged myeloid cells via inhibition of NF-κB signaling. **a** Prediction of candidate causal regulators of inflammaging based on Ingenuity Pathway Analysis (IPA). **b** Experimental design. Human PBMCs from young (Y, 21–28 years) and aged (A, 52–86 years) donors were treated with thymulin (T) or vehicle (V) for 24 h (top). Young and aged mice were treated daily with vehicle or thymulin by intraperitoneal injection for 1 week (center). Bone marrow-derived macrophages (BMDMs) from young or aged mice were differentiated with vehicle or thymulin (bottom). **c** Frequency of cytokine-producing CD33^+^CD11b^+^ PBMCs following ex vivo treatment with vehicle or thymulin for 24 h (*n* = 14 donors per group). **d**, **e** Frequency of cytokine-producing cells within CD11b^+^ blood cells from aged mice treated with vehicle or thymulin (*n* = 15 per group), shown as quantified data (**d**) with representative Boolean analysis (**e**). **f**, Heatmap showing the frequency of cytokine-producing cells within CD45⁺ blood cells from young and aged mice treated with vehicle or thymulin. **g** Representative UMAP image of CD45^+^ blood cells from young and aged mice treated with vehicle or thymulin. **h** Cytokine levels in supernatants from BMDMs derived from young and aged mice, measured by ELISA (young, *n* = 10; aged, *n* = 6). **i** NF-κB p65 DNA-binding activity in aged BMDMs (*n* = 6 per group). Exact *p*-values are provided in the Source Data. **j** Immunoblot analysis of phosphorylated IκBα in aged BMDMs stimulated with LPS. Representative images from two independent experiments with similar results. **k** Luciferase reporter assays. RAW264.7 cells were transfected with wild-type (WT) or NF-κB binding site mutant (Mut) *Il6* promoter constructs and stimulated with LPS in the presence of vehicle or thymulin. Data shown are from a representative experiment performed with duplicate technical replicates. The experiment was repeated independently three times with similar results. Statistical significance was assessed using paired (**c**, **h**) or unpaired (**d**) two-tailed *t*-test. ****p < 0.0001 by one-way ANOVA with Tukey’s multiple comparisons (**i**). Two-way ANOVA with Bonferroni’s multiple comparisons test (**k**). Mean ± SEM. Mo: monocytes, Gr: granulocytes, Mp: macrophages, DC: dendritic cells, CD4T: CD4⁺ T cells, CD8T: CD8⁺ T cells, B: B cells. *n* values represent independent donors (**c**), mice (**d**), or biological replicates from independent mice (**h**, **i**).

These findings indicate that non-bone marrow-derived circulating factors in young hosts suppress age-associated myeloid cell inflammatory activation, prompting us to identify the tissue sources and molecular mediators responsible for this effect. To this end, we performed Ingenuity Pathway Analysis (IPA) to identify candidate upstream regulators of the pro-inflammatory state associated with aging based on curated causal relationships (Fig. 6a and Supplementary Dataset 3). Among 163 aging-related molecules in the database, nine were identified as causal factors whose altered activity has been linked to the modulation of lifespan. Of these, genetic deficiency of *FOXO3*, *LMNA*, or *SIRT* family genes has been reported to increase expression of pro-inflammatory cytokines, including IL-1, IL-6, and TNF-α^28–30^. Notably, thymic atrophy is a shared phenotype in these knockout models^31–33^, suggesting a potential role for the thymus in regulating age-associated inflammation. We therefore hypothesized that thymus-derived circulating factors may contribute to the suppression of myeloid inflammation. Candidate thymic factors include thymosin α1, thymosin β4, thymulin, and thymopoietin^34^, which are produced by thymic epithelial cells. Among these, thymulin has been reported to suppress pro-inflammatory cytokine production in PBMCs in vitro^35^, and its circulating activity declines with age, becoming undetectable in older mice^36^. These observations prompted us to evaluate the anti-inflammatory effects of thymulin in vivo and in vitro (Fig. 6b).

Treatment of human PBMCs with thymulin ex vivo for 24 h reduced the expression of IL-1α, IL-1β, IL-6, and TNF-α in an age-dependent manner (Fig. 6c). In vivo, thymulin treatment for 1 week in aged mice resulted in a marked reduction in the frequency of circulating myeloid cells producing pro-inflammatory cytokines compared with vehicle-treated controls, whereas no significant effect was observed in young mice (Fig. 6d–g). Consistent with this, thymulin attenuated pro-inflammatory cytokine production from aged but not young bone marrow-derived macrophages following LPS stimulation (Fig. 6h).

To investigate the underlying mechanism, we focused on NF-κB signaling, a central regulator of pro-inflammatory cytokine expression with binding sites in the promoters of these genes^37^. Thymulin treatment inhibited NF-κB p65 DNA-binding activity in nuclear extracts from LPS-activated bone marrow-derived macrophages of aged mice (Fig. 6i) and decreased LPS-induced phosphorylation of IκBα (Fig. 6j), indicating inhibition of NF-κB activation. Next, we employed gain- and loss-of-function approaches using a luciferase reporter assay to evaluate the role of NF-κB signaling in thymulin-mediated suppression of myeloid cell pro-inflammatory cytokines. Thymulin inhibited LPS-induced NF-κB–dependent reporter activity, whereas mutation of the NF-κB binding site abolished this inhibitory effect (Fig. 6k). Collectively, these results demonstrate that thymulin suppresses pro-inflammatory cytokine production in aged myeloid cells through inhibition of NF-κB signaling.

### Thymulin suppresses tumor growth and renders tumors responsive to anti-PD-L1 therapy in an age-dependent manner

**Fig. 7:**
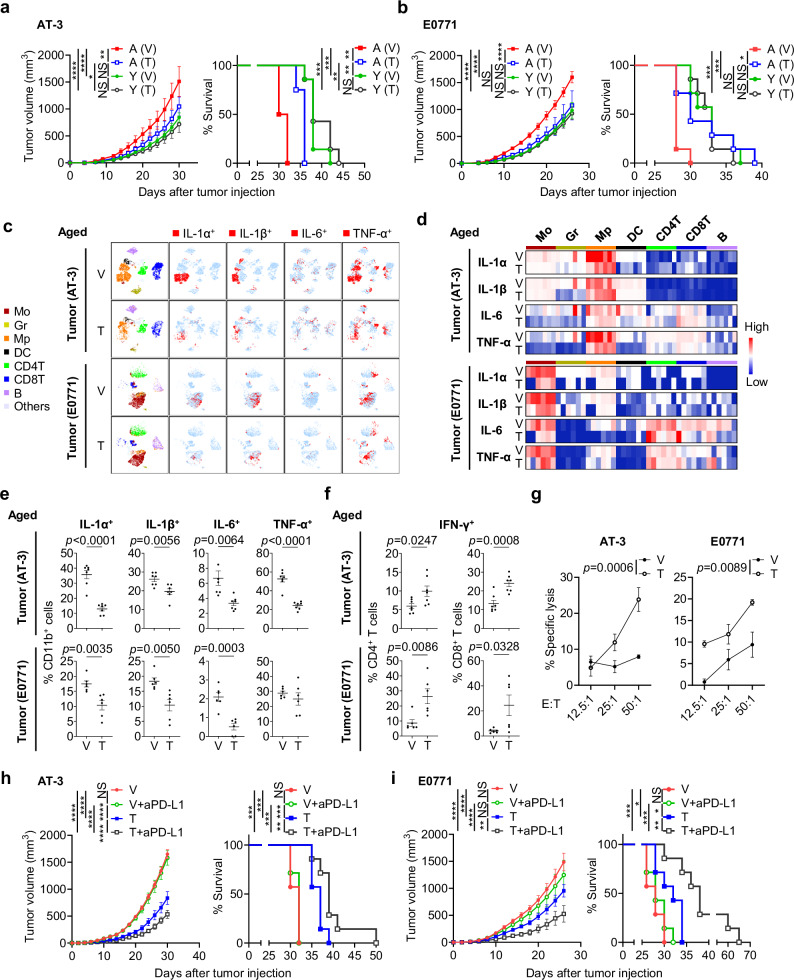
Thymulin suppresses tumor growth and renders tumors responsive to anti-PD-L1 therapy in aged mice. **a**, **b** Tumor growth curves (mean) and survival curves of AT-3 (**a**, young, *n* = 7 per group; aged, *n* = 4 per group) and E0771 (**b**, *n* = 7 per group) tumor-bearing mice treated with vehicle or thymulin. **c**–**f** Flow cytometric analysis of AT-3 (*n* = 7 per group) or E0771 (*n* = 6 per group) tumor-infiltrating immune cells in aged mice treated with vehicle or thymulin. **c** Representative UMAP image of tumor-infiltrating CD45^+^ cells. **d** Heatmap showing the frequency of pro-inflammatory cytokine (IL-1α, IL-1β, IL-6, and TNF-α)-producing cells among CD45⁺ cells infiltrating AT-3 or E0771 tumors. **e** Frequencies of pro-inflammatory cytokine-producing cells among CD11b⁺ cells infiltrating AT-3 or E0771 tumors. **f** Frequencies of IFN-γ-producing CD4⁺ or CD8^+^ T cells within AT-3 or E0771 tumor-infiltrating CD45^+^ cells. **g** Cytotoxic T lymphocyte (CTL) killing assay. Target tumor cells (AT-3 or E0771) were co-cultured with effector cells at indicated effector-to-target (E:T) ratios (12.5:1, 25:1, and 50:1) in the presence of thymulin or vehicle (*n* = 3 per group). **h**, **i** Tumor growth curves (mean, left) and survival curves (right) of AT-3 (**h**) and E0771 (**i**) tumor-bearing mice treated with vehicle, thymulin, and/or anti-PD-L1 antibody (*n* = 7 per group). Statistical significance was assessed using two-tailed *t*-test (**e**, **f**). *p < 0.05; **p < 0.01; ***p < 0.001; ****p < 0.0001 by two-way ANOVA with Bonferroni’s multiple comparisons test (tumor growth, CTL killing assay) and log-rank test (survival) (**a**, **b**, **g**, **h**, **i**); NS: not significant. Mean ± SEM. A: aged mice, Y: young mice, V: vehicle, T: thymulin. Mo: monocytes, Gr: granulocytes, Mp: macrophages, DC: dendritic cells, CD4T: CD4⁺ T cells, CD8T: CD8⁺ T cells, B: B cells. *n* values represent independent mice. Exact *p*-values are provided in the Source Data (**a**, **b**, **h**, **i**).

We next evaluated the in vivo efficacy of thymulin in tumor-bearing mice. Thymulin markedly delayed the growth of AT-3 and E0771 tumors and improved survival in aged mice, while having minimal effect in young mice (Fig. 7a, b). Similar age-dependent therapeutic effects were observed in BALB/c mice bearing EMT6 mammary adenocarcinoma (Supplementary Fig. 11a–e). Consistent with these findings, thymulin reduced the frequency of tumor-infiltrating myeloid cells producing pro-inflammatory cytokines, IL-1α, IL-1β, and IL-6, in an age-dependent manner (Fig. 7c–e and Supplementary Fig. 12a–c). Furthermore, thymulin treatment increased IFN-γ production by tumor-infiltrating CD4^+^ and CD8^+^ T cells in aged mice (Fig. 7f and Supplementary Fig. 12d) with enhanced cytotoxic T lymphocyte activity in splenocytes, as measured by target-specific lysis assays (Fig. 7g). Given that aged mice exhibit reduced responsiveness to immune checkpoint blockade^2,38^, we next tested whether thymulin could enhance the efficacy of anti-PD-L1 therapy. While anti-PD-L1 monotherapy had no impact on tumor growth and survival in aged mice, thymulin treatment rendered tumors responsive to anti-PD-L1 therapy, resulting in enhanced tumor control and prolonged survival in an age-dependent manner (Fig. 7h, i and Supplementary Fig. 12e). Together, these findings identify thymulin as a key regulator of age-associated inflammation that enhances antitumor immunity and improves responsiveness to immune checkpoint blockade in aged hosts.

## Discussion

Aging is accompanied by chronic low-grade inflammation that contributes to cancer progression and therapeutic resistance, yet the mechanisms driving this process and potential intervention strategies remain incompletely understood^22^. Here, we identify a marked expansion of circulating pro-inflammatory myeloid cells in aged mice and humans, characterized by increased production of IL-1α, IL-1β, IL-6, and TNF-α. These populations are further enriched in tumor-bearing aged mice and are associated with accelerated tumor progression in preclinical breast cancer models, while a similar age-associated pro-inflammatory myeloid profile is observed in human breast tumors. Using heterochronic parabiosis and bone marrow chimera approaches, we demonstrate that non-bone marrow-derived circulating factors in young hosts suppress age-associated myeloid cell inflammatory activation and tumor progression. Mechanistically, we identify thymulin as a thymus-derived factor that restrains inflammatory activation of aged myeloid cells through inhibition of canonical NF-κB signaling. Functionally, thymulin enhances antitumor immunity, increases IFN-γ-producing tumor-infiltrating T cells, delays tumor growth, and renders tumors responsive to anti-PD-L1 therapy in aged mice, highlighting its age-dependent immunomodulatory effects.

Chronic activation of the innate immune system is a hallmark of inflammaging, with monocytes and macrophages playing a central role^6,7,39^. Consistent with this, we observed increased frequencies of circulating pro-inflammatory myeloid cells in aged mice, with multiple subsets including monocytes, macrophages, and granulocytes showing elevated cytokine production. These subsets were further enriched in the tumor microenvironment, where intratumoral myeloid cells exhibited a pronounced pro-inflammatory phenotype. In the E0771 tumor model, monocytes displayed the highest expression of inflammatory cytokines, whereas in the AT-3 model, macrophages represented the predominant inflammatory myeloid population, indicating that the identity of the most pro-inflammatory subset is model-dependent. CD11b⁺Ly6C⁺Ly6G⁺ granulocytes/neutrophils were included in our analysis and showed comparable age-associated trends (Supplementary Figs. 2, 3b, 5c, d, 10 d). However, inflammatory signatures were more prominently enriched in monocyte/macrophage populations in the breast cancer models used in this study, consistent with observations in human breast tumors^9,21^.

To assess the contribution of tumor-induced inflammation, we evaluated changes in circulating pro-inflammatory myeloid cells before and after tumor implantation within the same animals (Supplementary Fig. 4). Tumor-bearing mice exhibited a modest increase in circulating pro-inflammatory myeloid cells in both young and aged cohorts, indicating that tumor presence contributes to systemic inflammation. However, the magnitude of this effect was substantially smaller than the difference observed between young and aged mice, demonstrating that aging is the key driver of the pro-inflammatory phenotype. Consistent with this, age-associated increases in cytokine-producing myeloid cells were observed across mouse strains (C57BL/6 and BALB/c), in both male and female mice, and in non-tumor-bearing settings, indicating that these alterations are not solely dependent on tumor-derived signals. Together, these findings support a model in which tumor-induced inflammation acts as a condition-dependent modulatory factor, whereas aging establishes a baseline pro-inflammatory state that shapes myeloid cell function.

The expansion of pro-inflammatory myeloid cells in aged mice has been attributed in part to hematopoietic skewing toward myeloid lineage commitment^17,40–42^. However, our findings indicate that this inflammatory state is also shaped by a decline in endogenous mechanisms that restrain myeloid cell inflammatory activation. Heterochronic parabiosis experiments demonstrate that age-associated dysregulation of pro-inflammatory states in circulating and intratumoral myeloid cells is partially reversible by factors present in young circulation, consistent with previous reports of rejuvenation of aged immunity^17,20,42^. Bone marrow and mixed bone marrow chimera experiments further demonstrate that this regulation is mediated predominantly by non-bone marrow-derived circulating factors. In mixed chimeras, both Y + A → Y and Y + A → A mice contain young and aged hematopoietic compartments, yet aged donor-derived myeloid cells exhibit distinct inflammatory phenotypes depending on host age, indicating a predominant influence of host-derived signals. Although contributions from bone marrow-derived factors cannot be fully excluded, these findings support an important role for non-bone marrow-derived circulating factors in regulating pro-inflammatory cytokine production in aged myeloid cells.

Notably, the influence of host age on myeloid cell inflammatory activation varies across biological settings. Young bone marrow-derived myeloid cells exhibited increased inflammatory potential when transferred into aged hosts (Y → A versus Y → Y; Supplementary Fig. 8c). In contrast, in mixed chimeras, cytokine production by young donor-derived myeloid cells was comparable between Y + A → Y and Y + A → A mice in peripheral blood, likely reflecting modulation of cell-intrinsic responses by coexisting hematopoietic compartments. However, within the aged tumor microenvironment, young donor-derived myeloid cells acquired a more pro-inflammatory phenotype, indicating that tissue environment and host age jointly regulate inflammatory activation. These findings suggest a hierarchical regulation in which the tumor microenvironment has a greater impact than the circulation. Delayed tumor progression and reduced myeloid cell inflammatory activation in Y + A → Y chimeras compared with Y + A → A mice may in part reflect differences in thymic lymphopoietic function. However, using Rag2^⁻/⁻^ heterochronic mixed bone marrow chimeras, we demonstrate that non-bone marrow-derived circulating factors from young hosts attenuate age-associated myeloid cell inflammatory activation independent of adaptive immunity. Notably, accelerated tumor progression was observed in aged hosts even in the absence of T and B cells, indicating that aged myeloid cells are sufficient to promote tumor progression.

Ingenuity Pathway Analysis identified the thymus as a potential upstream regulator of age-associated inflammation. While it remains unclear whether complete thymic absence, as observed in *Foxn1*^*nu*^ (nude) mice, fully recapitulates inflammaging, age-associated decline in *Foxn1* expression contributes to thymic dysfunction and has been linked to increased systemic inflammatory cytokines, including IL-6 and IL-1β^43^. Consistent with this, clinical studies demonstrate a close association between thymic function and systemic inflammation. Thymectomy in adults has been associated with elevated pro-inflammatory cytokines, increased cancer risk, and higher mortality^44^, while independent studies show that thymic health correlates with chronic inflammation and responses to immunotherapy across multiple cancer types, including breast cancer^45,46^. Together, these findings support a model in which age-related thymic decline contributes to inflammaging and shapes cancer susceptibility and therapeutic response.

Efforts to identify circulating factors that counteract inflammaging have implicated multiple processes, including oxidative stress, DNA damage, impaired autophagy, microbiota changes, and cellular senescence^7,22^. However, few studies have shown reversal of age-related chronic inflammation in the context of tumor immunity. In this study, thymulin reduced pro-inflammatory cytokine-producing myeloid cells, delayed tumor progression, and restored responsiveness to anti-PD-L1 therapy in aged mice. These findings support the concept that inflammaging contributes to cancer progression and that targeting inflammatory pathways can improve antitumor immunity. Our results showing the enhanced adaptive immunity in the form of increased IFN-γ production in tumor-infiltrating T cells also align with recent evidence that reversal of the chronic inflammation restores T-cell function^12,13,18^. Importantly, the immunomodulatory effects of thymulin are age-dependent. Thymulin treatment had minimal effects in young mice and did not enhance antitumor immunity or responsiveness to anti-PD-L1 therapy, whereas robust effects were observed in aged hosts. This suggests that thymulin primarily acts by restoring age-associated immune dysfunction rather than broadly enhancing immune activation. These findings may have translational relevance, as aging is associated with reduced responsiveness to immunotherapy, and thymulin may help restore immune competence in older patients.

Thymulin is a thymus-derived nonapeptide (H-Pyr-Ala-Lys-Ser-Gln-Gly-Gly-Ser-Asn-OH) whose circulating levels decline with age^34,36^. Plasma thymulin levels peak during childhood and decrease progressively after adolescence^36,47^. Thymulin levels are also altered under pathological immune conditions, including autoimmune diseases and immunodeficiency, and have been reported to be reduced in cancer patients compared with age-matched healthy controls^48,49^. Previous studies have shown that thymulin suppresses pro-inflammatory cytokine production and inhibits NF-κB signaling^35,50^. Consistent with this, our data indicate that thymulin suppresses pro-inflammatory cytokine production in aged myeloid cells through inhibition of NF-κB activation. The transcription factor, NF-κB, plays a critical role in responding to various types of stimuli, upregulates the transcription of inflammatory cytokines upon activation, and is implicated in inflammaging and many of the hallmarks of aging^7,37^.

While murine models have been instrumental for studying immune regulation and therapeutic responses, their translational relevance must be interpreted with caution. Our work described herein aligns with the prevailing notion that using young mice could underestimate the impact of chronic inflammation in cancer, limiting the evaluation of novel treatment modalities on antitumor immune responses^2,51^. Given that the immune system undergoes substantial remodeling with age, the use of aged mice may better capture key features of human disease and improve the predictive value of preclinical immunotherapy studies. It is well established that immune composition and inflammatory responses can vary across mouse strains. Although we observed comparable age-associated myeloid cell inflammatory activation in C57BL/6 and BALB/c mice, further validation in additional models would strengthen this conclusion. Finally, as our preclinical studies were conducted in breast cancer models (AT-3 and E0771), these findings should be interpreted within this disease context. While our results highlight a mechanism by which systemic factors, particularly thymulin, regulate age-associated inflammation and tumor progression, the extent to which these findings generalize to other cancer types remains to be determined.

In summary, our findings identify an unexpected role for the thymus in regulating age-related myeloid inflammation through thymulin. These results suggest that age-related thymic involution not only impairs adaptive immunity but also promotes myeloid-driven pro-inflammatory innate responses, thereby contributing to tumor progression and therapeutic resistance.

## Methods

### Ethics statement

All animal experiments were performed in accordance with and approved by the Institutional Animal Care and Use Committee of the University of Southern California. The maximal tumor size permitted by the approved animal protocol was 15 mm in diameter. Mice were euthanized when tumors reached this limit or when they became moribund and showed signs of cachexia, lateral recumbency, observable weight loss, or lack of response to noxious stimuli. The maximal tumor burden permitted by the institutional guidelines was not exceeded in any experiment.

### Mice

Female and male CD45.1 or CD45.2 C57BL/6 mice, as well as BALB/c mice, were obtained from Charles River Laboratories or The Jackson Laboratory. Rag2 knockout (Rag2^–/–^; B6.Cg-*Rag2*^tm1.1Cgn^/J) mice were obtained from the Jackson Laboratory and were bred in-house. Mice were maintained under specific pathogen-free conditions according to institutional guidelines. Based on established lifespan comparisons, mice at 8–12 weeks of age are generally considered young adults, corresponding to early adulthood in humans (approximately 20–30 years). In contrast, mice at 65–75 weeks are commonly used to represent a late-life or aging stage, broadly analogous to late middle to early elderly stages in humans (approximately 50–70 years). However, such cross-species comparisons are inherently non-linear and depend on the physiological parameter assessed^52^. In this study, C57BL/6 mice aged 8–12 weeks and 65–75 weeks were used as the young and aged cohorts, respectively. For BALB/c mice used in this study, animals aged ≥65 weeks were defined as aged based on experimental availability.

### Cell lines

The E0771 cell line was purchased from the CH3 BioSystems. B16-F10 (B16), RAW 264.7, and EMT6 cell lines were purchased from the American Type Culture Collection (ATCC). The AT-3 cell line was a gift from Dr. Scott Abrams (Roswell Park Comprehensive Cancer Center, Buffalo, NY). E0771, B16, and RAW 264.7 cells were cultured in RPMI 1640 (Gibco) supplemented with 10% FBS (Sigma-Aldrich), 1% nonessential amino acid (NEAA; Gibco), 2 mmol/L L-glutamine (Gibco), 0.5% penicillin/streptomycin (Gibco), and 50 µmol/L 2-mercaptoethanol (Gibco). AT-3 and EMT6 cells were cultured in DMEM (Gibco) supplemented with 10% FBS, 1% NEAA, 2 mmol/L L-glutamine, 0.5% penicillin/streptomycin, and 50 µmol/L 2-mercaptoethanol. These cell lines were authenticated by morphology, phenotype, and growth, and routinely screened for *Mycoplasma* and maintained at 37 °C in a humidified 5% (E0771, B16, RAW 264.7, EMT6) or 7% (AT-3) CO_2_ atmosphere.

### Plasmids

PmIL-6 FL and pmIL-6 mut NF-κB were gifts from Dr. Gail Bishop (Addgene plasmid # 61286; http://n2t.net/addgene:61286; RRID:Addgene_61286 and Addgene plasmid # 61293; http://n2t.net/addgene:61293; RRID:Addgene_61293, respectively)^53^.

### Tumor model

E0771 (5 × 10^5^), AT-3 (5 × 10^5^), or EMT6 (2 × 10^5^) tumors were surgically implanted under anesthesia with isoflurane into the fourth mammary gland of female mice as described^54,55^. B16 (5 × 10^5^) tumor cells were injected subcutaneously on the left flank of male mice. Tumor growth was measured 3–5 times a week, and the volumes were calculated by determining the length of short (l) and long (L) diameters (volume = *l*^2^ × *L*/2) as previously described^54,55^.

### In vivo treatment

For in vivo thymulin-conditioning, thymulin solution (1.5 mg/kg of body weight) was prepared from thymulin acetate (MedChemExpress, Monmouth Junction, NJ, USA) with an equimolar concentration of ZnCl_2_ (39059, Sigma-Aldrich, St. Louis, MO, USA) in PBS (Gibco), and was injected intraperitoneally (i.p.) starting on day 1 after tumor cell injection and continued daily for 4 weeks. Control mice received ZnCl_2_ in PBS (vehicle). For PD-L1 blockade, anti-PD-L1 Ab (clone 10 F.9G2, BioXcell) was given i.p. on day 7, 10, 13, 16, 19, 22 after the tumor cell inoculation at a dose of 200 μg/mouse^54,55^.

### Flow cytometry

Single-cell suspensions of mouse peripheral blood and tumors were prepared for flow cytometric analysis. Red blood cells in blood were lysed using ACK Lysis Buffer (Life Technologies). Tumor tissues were weighed, minced, filtered through 70-μm filters, and stored at −80 °C in FBS (Hyclone, Waltham, MA) with 10% DMSO (Sigma) before analysis. Cells were blocked with anti-mouse CD16/32 (553142, BD Biosciences, San Diego, CA, USA) and surface-stained with indicated markers. Live/dead cell discrimination was performed using LIVE/DEAD Fixable Near-IR Dead Cell Stain Kit (Life Technologies). To evaluate changes in the myeloid and lymphoid compartments of peripheral blood and the tumor microenvironment, we used the 23-color flow cytometry gating strategy (Supplementary Fig. 1) as described previously^54^. For analysis of cytokine expression, intracellular IL-1α, IL-1β, IL-6, TNF-α, and IFN-γ assays were performed using Fixation/Permeabilization Solution Kit (BD Biosciences) according to the manufacturer’s recommendations. Prior to intracellular staining, cells were stimulated ex vivo for 4 h at 37 °C in stimulation media composed as follows: RPMI 1640 medium with 10% FBS (Hyclone), 50 μg/ml of gentamicin (Gibco), 25 mM HEPES (Gibco), 2 mM L-glutamine (Gibco), 0.1 mM sodium pyruvate (Gibco), 0.1 mM NEAA (Gibco), LPS (100 ng/ml; LPS-SM ultrapure, InvivoGen), and 1× Cell Stimulation Cocktail with protein transport inhibitors (containing phorbol 12-myristate 13-acetate (PMA), ionomycin, brefeldin A, and monensin; eBioscience, Thermo Fisher Scientific, Waltham, MA). Antibodies used in this study are listed in Supplementary Table S1 and were diluted using Brilliant Stain Buffer (566349, BD Biosciences). Samples acquired on Aurora (Cytek, San Diego, CA) cytometers were analyzed with FlowJo software (Treestar). Visualization of the multidimensional data was performed using the dimensionality reduction algorithm Uniform Manifold Approximation and Projection (UMAP), provided in FlowJo Plugin Exchange (v4.0.4, Treestar). Annotated pie charts were generated by SPICE software developed by the National Institutes of Health (v6.1).

### Parabiosis

Parabiosis surgery was performed as previously described^56^. In brief, mirror-image skin incisions were made from the elbow to the knee in each mouse anesthetized with isoflurane. The peritoneal openings of the adjacent parabionts were sutured together. Forelimbs and hindlimbs were sutured together, and the skin of each incision was closed using continuous suture. Each mouse was injected subcutaneously with buprenorphine, as directed, for pain and monitored during recovery. Parabiosed pairs were analyzed more than 2 weeks after surgery.

### Generation of bone marrow chimeras

Bone marrow and mixed bone marrow chimeras were generated as previously described^54,57^. First, recipient C57BL/6 mice were irradiated with 500 cGy followed by a second dose of 550 cGy 3 h apart. To obtain donor bone marrow, femurs and tibiae were harvested, and the bone marrow was flushed out. For bone marrow chimeras, 1 × 10^7^ bone marrow cells from aged or young mice were injected into irradiated young or aged mice. For mixed bone marrow chimeras, 1 × 10^7^ bone marrow cells from young and aged mice of a 1:1 mixture were injected into irradiated young or aged mice. After 8–12 weeks, the recipients were used for the experiments.

### Generation of bone marrow-derived macrophages

Bone marrow-derived macrophages were generated as described previously^58^. Briefly, bone marrow cells from flushed marrow cavities of femurs and tibiae of >65 weeks old female C57BL/6 mice were cultured in RPMI 1640 with 10% FBS containing 10 ng/ml macrophage colony-stimulating factor (M-CSF, PeproTech, Rocky Hill, NJ, USA) with/without thymulin (1 µg/ml) and an equimolar concentration of ZnCl_2_. A control group was cultured in the same media with M-CSF and ZnCl_2_. Medium was changed daily. One week later, bone marrow-derived macrophages were harvested. Cells were stimulated with LPS (100 ng/ml; LPS-SM ultrapure, InvivoGen, San Diego, CA, USA) for 24 h before ELISA analysis.

### ELISA

Levels of IL-1α, IL-1β, IL-6, and TNF-α secreted by aged bone marrow-derived macrophages were measured using ELISA MAX Deluxe Set Mouse IL-1α (BioLegend), Mouse IL-1β ELISA Kit (Sigma-Aldrich), Quantikine Mouse IL-6 ELISA Kit (R&D Systems), and BD OptEIA Mouse TNF ELISA Set II (BD Biosciences), respectively. The absorbance of each ELISA plate was measured using a microplate reader CLARIOstar Plus (BMG LABTECH, Weston Parkway, NC).

### NF-κB DNA-binding assay

The NF-κB p65 DNA-binding activity was measured with the TransAM NF-κB p65 according to the manufacturer’s recommendations (Active Motif, Carlsbad, CA, USA). Nuclear extracts were prepared from 1 × 10^6^ bone marrow-derived macrophages generated from aged mice. After cells are cultured with thymulin (1 μg/ml) and an equimolar concentration of ZnCl_2_ or ZnCl_2_ only (control) for 1 week, the cells were activated with LPS (100 ng/ml) for 1 h, and cell lysates were prepared in 200 μl lysis buffer [10 mM Hepes-KOH, pH 7.8, 10 mM KCl, 0.1 mM EDTA, pH 8.0, protease inhibitor mixture (Roche), and 0.1% Nonidet P-40], and nuclear extracts were obtained in 30 μl buffer [50 mM Hepes-KOH, pH 7.8, 420 mM KCl, 0.1 mM EDTA, pH 8.0, 5 mM MgCl_2_, protease inhibitor mixture (Roche), and 20% glycerol]^58^. Nuclear extracts (5 μl) in microwells were used to evaluate NF-κB DNA-binding activity.

### Immunoblot analysis

Cells were lysed with 200 μl lysis buffer [10 mM Hepes-KOH, pH 7.8, 10 mM KCl, 0.1 mM EDTA, pH 8.0, protease inhibitor mixture (Roche), and 0.1% Nonidet P-40]. The cell lysates were separated by standard SDS-PAGE (Bioland Scientific, Paramount, CA) and analyzed by immunoblotting using the Trans-Blot Turbo Transfer System (Bio-Rad, Hercules, CA, USA). The following antibodies were used: anti-phospho-IκBα (Ser32/36; 5A5, Cell Signaling Technology), goat anti-mouse IgG-HRP (Poly4053, BioLegend), and anti-β-actin-HRP (2F1-1, BioLegend). The dilution ratios for anti-phospho-IκBα, goat anti-mouse IgG-HRP, and anti-β-actin-HRP were 1:1000, 1:2000, and 1:1000, respectively. The Western HRP Substrate (SuperSignal West Pico PLUS Chemiluminescent Substrate, Thermo Fisher Scientific) was used for the development of positive signals, and chemiluminescence was detected using an iBright 1500 (Thermo Fisher Scientific).

### Luciferase reporter assay

Luciferase reporter assays were performed using the Dual-Luciferase Reporter Assay System (Promega, Madison, WI, USA). For transfections, 1 × 10^5^ RAW 264.7 cells were transfected with 0.8 μg of pmIL-6 FL (WT NF-κB) or pmIL-6 mut NF-κB plasmid (mut NF-κB), and 0.1 μg of pRL-TK using Lipofectamine 2000 (Invitrogen) and then plated in a 24-well plate at 1 × 10^5^ cells/well. After 24 h, cells were activated by LPS (100 ng/ml, InvivoGen) and thymulin (1 μg/ml) with an equimolar concentration of ZnCl_2_ or ZnCl_2_ only (control) for 24 h. Following activation, cell extracts were prepared using Passive Lysis Buffer (Promega). Luciferase activity was determined from a 20-μl cell extract and measured on the CLARIOstar Plus (BMG LABTECH) microplate reader.

### CTL killing assay

CTL killing assays were performed as previously described^59^. Cytotoxicity was assessed using a 7-AAD/CFSE cell-mediated cytotoxicity assay kit (Cayman Chemical, MI, USA) according to the manufacturer’s instructions. Briefly, whole splenocytes were isolated from AT-3 or E0771 tumor-bearing aged mice 2 weeks after tumor implantation, following in vivo thymulin or vehicle (control) conditioning as described above. A total of 4 × 10⁷ splenocytes were co-cultured with 2 × 10⁶ IFN-γ-treated (100 U/ml; PeproTech) AT-3 or E0771 tumor cells in the presence of thymulin (1 μg/ml) and an equimolar concentration of ZnCl₂ or ZnCl₂ alone (control). After 5 days, cells were harvested and used as effector cells (E). For target cell (T) preparation, AT-3 or E0771 tumor cells were treated with IFN-γ (100 U/ml) for 48 h prior to use. Target cells were labeled with CFSE (5 mM stock solution in PBS) for 10 min at 37 °C, washed twice with PBS, and immediately used in the assay. CFSE-labeled target cells were co-incubated with effector cells at the indicated effector-to-target (E:T) ratios (12.5:1, 25:1, and 50:1) for 4 h. Flow cytometric analysis was performed as described above. The percentage of specific lysis was calculated as follows: % specific lysis = 100 × (% sample lysis − % basal lysis) / (100 − % basal lysis), where % sample lysis and % basal lysis represent the percentage of CFSE⁺ 7-AAD⁺ target cells in the presence or absence of effector cells, respectively.

### Human PBMC analysis

Written informed consent was obtained from 93 healthy donors aged between 21 and 87 with no personal history of cancer for the collection, storage, and analysis of blood samples under the Institutional Review Board of the University of Southern California (approval number: HS-22-00354) in accordance with the Declaration of Helsinki. Peripheral blood was obtained, and PBMCs were isolated using Lymphocyte Separation Medium (Corning) density gradient centrifugation and stored as previously described^60–63^. For the evaluation of the effect of thymulin, cells were cultured with thymulin (1 μg/ml) and an equimolar concentration of ZnCl_2_ or ZnCl_2_ only (control) for 24 h and were activated by LPS (100 ng/ml, InvivoGen) and 1× Cell Stimulation Cocktail with protein transport inhibitors (Thermo Fisher Scientific) for 4 h and were analyzed by flow cytometry.

### IPA database exploration

To identify candidate factors associated with aging and inflammation, we performed a systematic exploration of the QIAGEN Ingenuity Pathway Analysis (IPA) database^64^. Within the “Diseases and Functions” category, the term “Aging” was used as a query. This search initially yielded 163 molecules, all of which were extracted for further analysis (Supplementary Dataset 3). These molecules were then filtered based on the “Causal or Correlated” annotation provided in IPA, and 35 molecules classified as “Causal” were selected. Among these, we further narrowed down the candidates by applying the criteria “Disease or Function = Longevity” and “Effect on Disease or Function = decreases/affects,” resulting in 9 molecules. Finally, from these 9 candidates, we identified molecules for which knockout mouse models have been reported to exhibit increased levels of pro-inflammatory cytokines, including IL-1, IL-6, and/or TNF-α. Based on this criterion, three factors—*FOXO3*, *LMNA*, and *SIRT* family—were selected for subsequent analyses.

### Single-cell RNA sequencing

Count matrices, barcodes, feature data, and patient metadata for the eight oldest and eight youngest untreated primary breast tumors were downloaded from the Gene Expression Omnibus (GEO) (GSE176078). Cells with more than 200 detected genes (nFeature_RNA > 200), more than 250 total detected transcripts (nCount_RNA > 250), and less than 20% mitochondrial content were retained for downstream analyses. Doublets were removed using the DoubletFinder R package (v2.0.6)^65^. Briefly, each patient sample is preprocessed using Seurat (v5.4.0), and doublet detection was performed using the top 50 principal components. Artificial doublets were generated and integrated into each patient dataset at a defined proportion (pN = 0.25). The optimal neighborhood parameter (pK) was determined empirically using the *paramSweep, summarizeSweep, and find.pK* functions. Optimal pK was selected based on the maximum BCmetric score. The expected number of doublets (nExp) was estimated as 7.5% of the total cell number and adjusted for homotypic doublets based on cluster identities. Cells were then classified using DoubletFinder, and predicted doublets were removed from downstream analyses. Filtered datasets were merged, normalized using SCTransform, and subjected to principal component analysis (PCA) for dimensionality reduction. The *ElbowPlot* function was used to determine the optimal dimensionality of the dataset (PCs = 30). Data integration was performed using Seurat’s *IntegrateLayers* function with canonical correlation analysis (CCA) to minimize batch effects. A shared nearest neighbor graph was constructed using the *FindNeighbors* function, and cell clustering was performed using the *FindClusters* function at a resolution of 0.8. Differentially expressed genes (>25% cells, >0.25-fold change) were identified using the *FindAllMarkers* function and used for cluster annotation. Differential expression between annotated clusters was determined using the MAST method via the *FindMarkers* function, using a minimum expression proportion of 25% and a minimum fold change of 0.1. Single-cell gene set enrichment analysis (GSEA) was performed using VISION (v3.0.2) to calculate enrichment scores for curated gene signatures and biological pathways.

### Statistical analysis

Statistical analysis was performed using a two-tailed Student’s *t*-test for comparisons between 2 groups, a one-way ANOVA with Tukey’s multiple comparisons test for comparisons among more than two groups, a two-way ANOVA with Bonferroni’s multiple comparisons test, the Mantel–Cox method (log-rank test) for survival analysis, or Pearson correlation analysis to assess relationships among variables, using GraphPad Prism 10.5.0 (GraphPad Software). *p* < 0.05 was considered statistically significant. Data are presented as mean ± SEM.

### Reporting summary

Further information on research design is available in the Nature Portfolio Reporting Summary linked to this article.

## Supplementary information

Supplementary Information

Description of Additional Supplementary Files

Supplementary Dataset 1

Supplementary Dataset 2

Supplementary Dataset 3

Reporting Summary

Transparent Peer Review file

## Source data

Source Data

## Data Availability

The publicly available scRNA-seq dataset analyzed in this study was obtained from the Gene Expression Omnibus (GEO) database under accession code GSE176078^21^ and is available at https://www.ncbi.nlm.nih.gov/geo/query/acc.cgi?acc=GSE176078. Source data generated in this study are provided with this paper.
