# Supplementary material for: Thymulin restrains age-associated myeloid inflammation and enhances cancer immunotherapy
Source: Nat Commun. 2026 Jul 21;17:6534. doi: 10.1038/s41467-026-75383-0 (PMC13389034; doi:10.1038/s41467-026-75383-0)
Supplement: Supplementary file 1 — Supplementary Information [file 41467_2026_75383_MOESM1_ESM.pdf]

Supplementary Figures and Tables

Thymulin restrains age-associated myeloid inflammation and enhances cancer immunotherapy

Hisashi Kanemaru[1], Steven Luong[1], Yuta Yamamoto[1], Yukari Mizukami[1], Fumito Ito[1, 2, *]

[1] Department of Surgery, Keck School of Medicine of the University of Southern California, Los Angeles, CA, USA

[2] Department of Immunology and Immune Therapeutics, Keck School of Medicine of the University of Southern California, Los Angeles, CA, USA

The PDF file includes:

Supplemental Figures 1-12

Tables S1

Supplementary Fig. 1

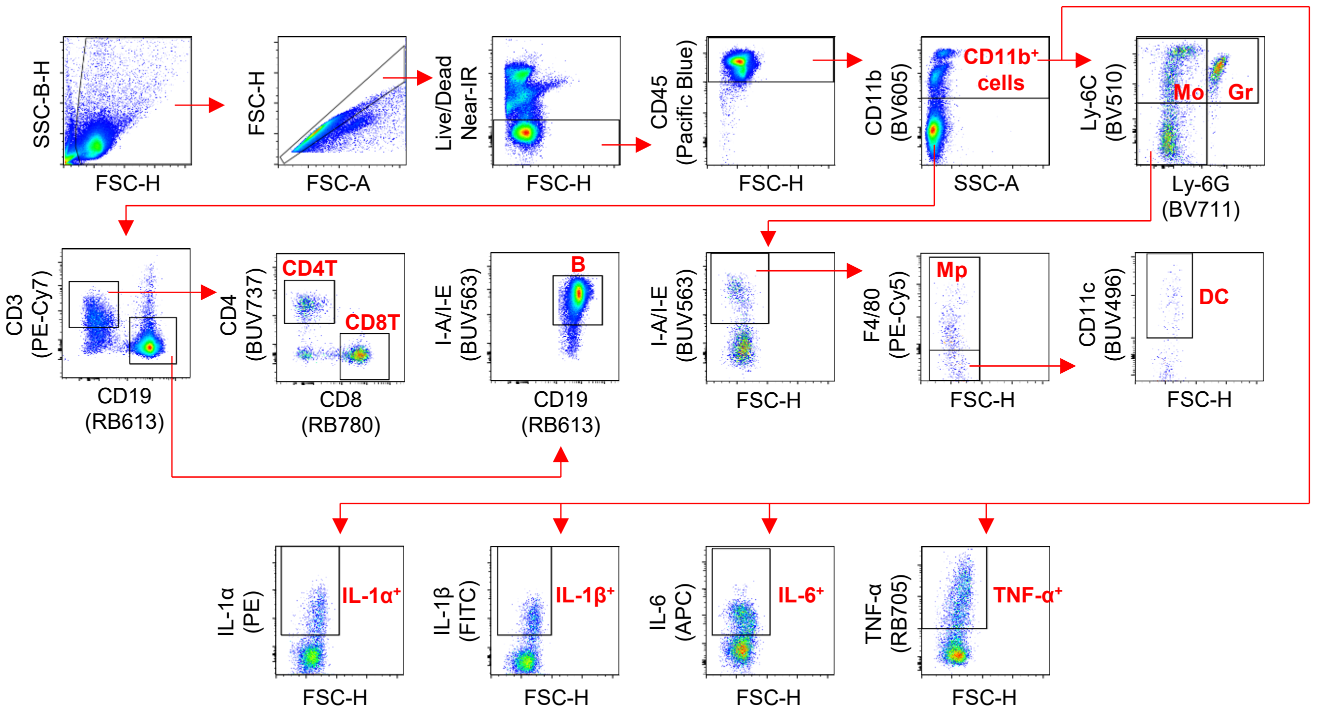

Supplementary Fig. 1. Gating strategy for identification of immune cell subsets and cytokine-producing CD11b^{+} cells.

Cells were first gated on singlets (FSC-H vs FSC-A) and live cells using Live/DEAD Fixable Near-IR stain, followed by selection of CD45^{+} leukocytes. Monocytes (Mo) were defined as CD11b^{+}Ly6C^{+}Ly6G^{-} cells; granulocytes (Gr) as CD11b^{+}Ly6C^{+}Ly6G^{+} cells; macrophages (Mp) as CD11b^{+}Ly6C^{-}Ly6G^{-}I-A/I-E^{+}F4/80^{+} cells; and dendritic cells (DC) as CD11b^{+}Ly6C^{-}Ly6G^{-}I-A/I-E^{+}F4/80^{-}CD11c^{+} cells. CD4^{+} T cells (CD4T) were defined as CD11b^{-}CD19^{-}CD3^{+}CD4^{+}CD8^{-} cells, CD8^{+} T cells (CD8T) as CD11b^{-}CD19^{-}CD3^{+}CD4^{-}CD8^{+} cells, and B cells (B) as CD11b^{-}CD3^{-}CD19^{+}I-A/I-E^{+} cells. Cytokine-positive gates were defined using fluorescence minus one (FMO) controls.

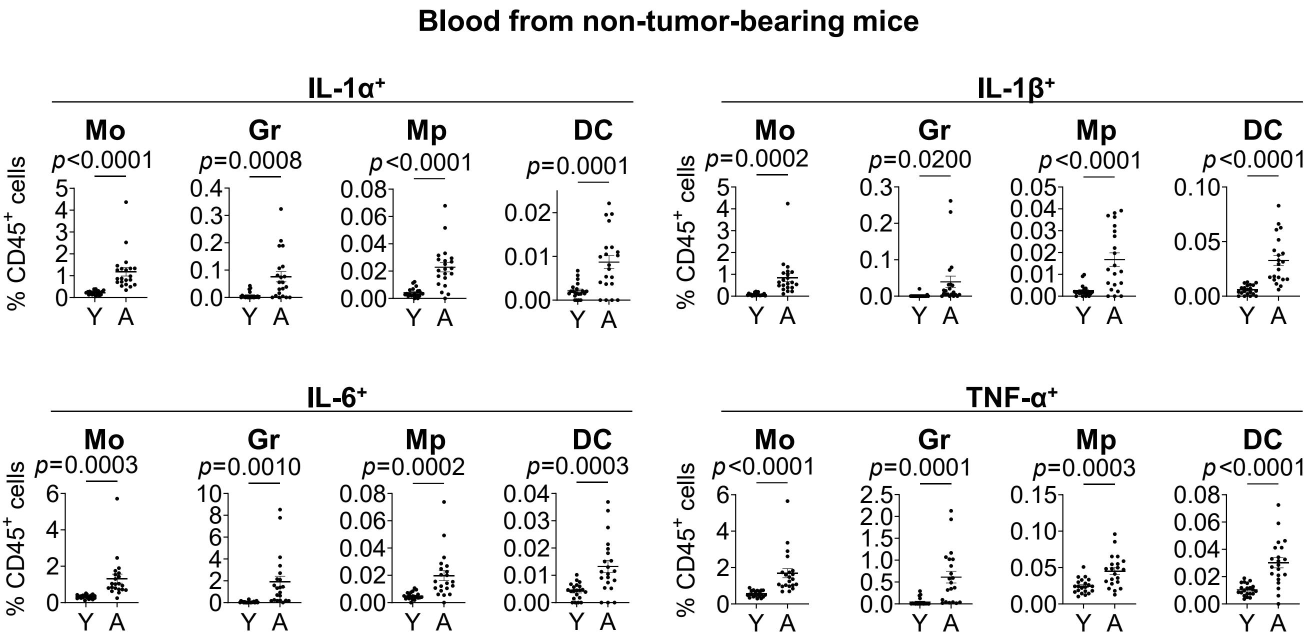

Supplementary Fig. 2. Pro-inflammatory cytokine-producing circulating myeloid cell subsets in young and aged female mice, related to Figure 1.

Frequency of cytokine-producing cells within $CD45^+$ blood cells from young (Y) and aged (A) C57BL/6 female mice without tumors (n = 21 per group).

Statistical significance was assessed using two-tailed t-tests. n values represent independent mice. Mean ± SEM. Mo, monocytes; Gr, granulocytes; Mp, macrophages; DC, dendritic cells; CD4T, $CD4^+$ T cells; CD8T, $CD8^+$ T cells; B, B cells.

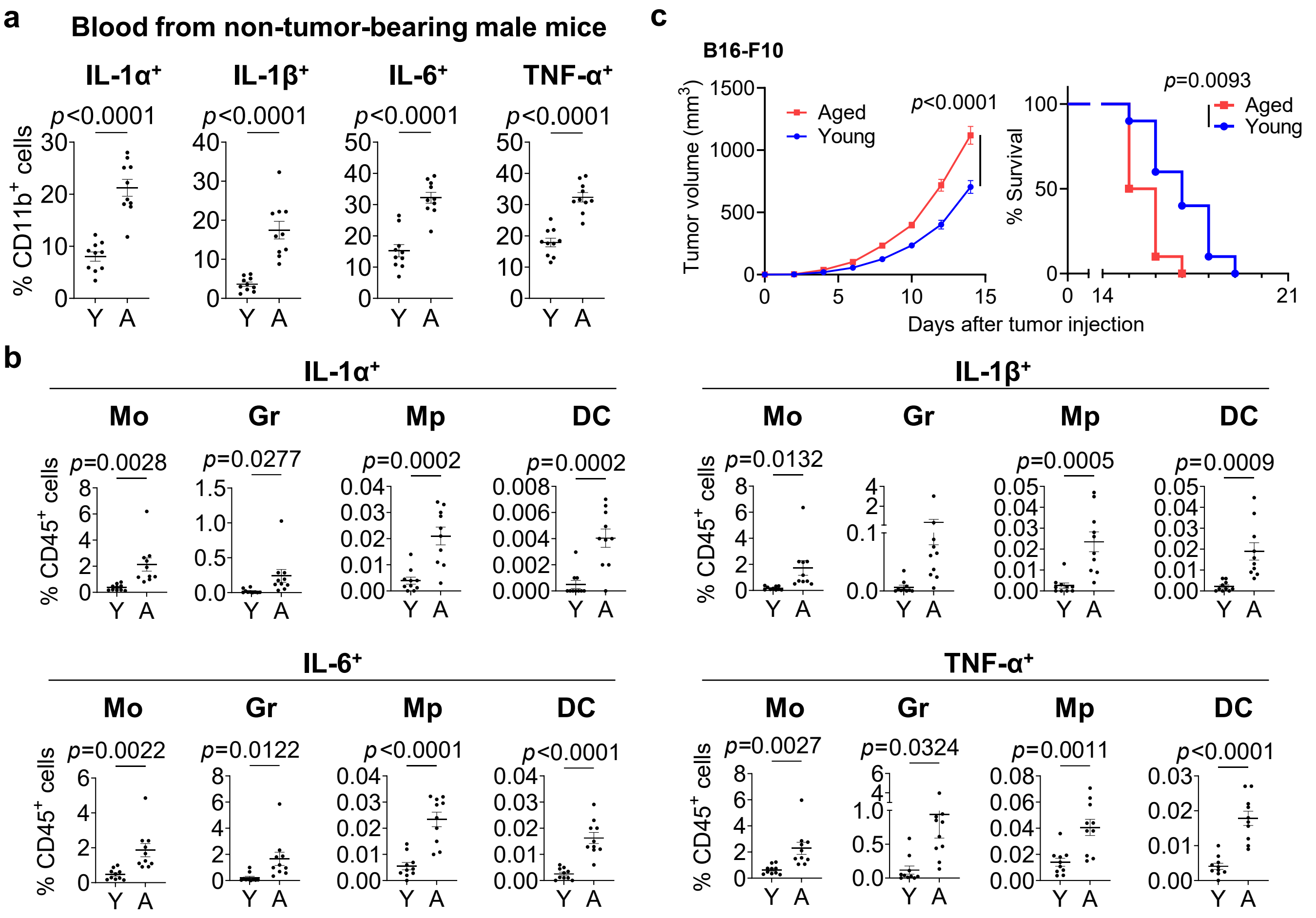

Supplementary Fig. 3. Circulating pro-inflammatory myeloid cells are increased in aged male mice and are associated with accelerated tumor progression, related to Figure 1.

a, **b** Frequency of cytokine-producing cells within CD11b$^+$ (**a**) or CD45$^+$ (**b**) blood cells from young (Y) and aged (A) male C57BL/6 mice without tumors (*n* = 10 per group).

c Tumor growth curves (mean, left) and survival curves (right) of male C57BL/6J mice bearing B16-F10 tumors (*n* = 10 per group).

Statistical significance was assessed using two-tailed *t*-tests (**a**, **b**), two-way ANOVA with Bonferroni's multiple comparisons test (tumor growth) and log-rank test (survival) (**c**). Mean ± SEM. *n* values represent independent mice. Mo, monocytes; Gr, granulocytes; Mp, macrophages; DC, dendritic cells; CD4T, CD4$^+$ T cells; CD8T, CD8$^+$ T cells; B, B cells.

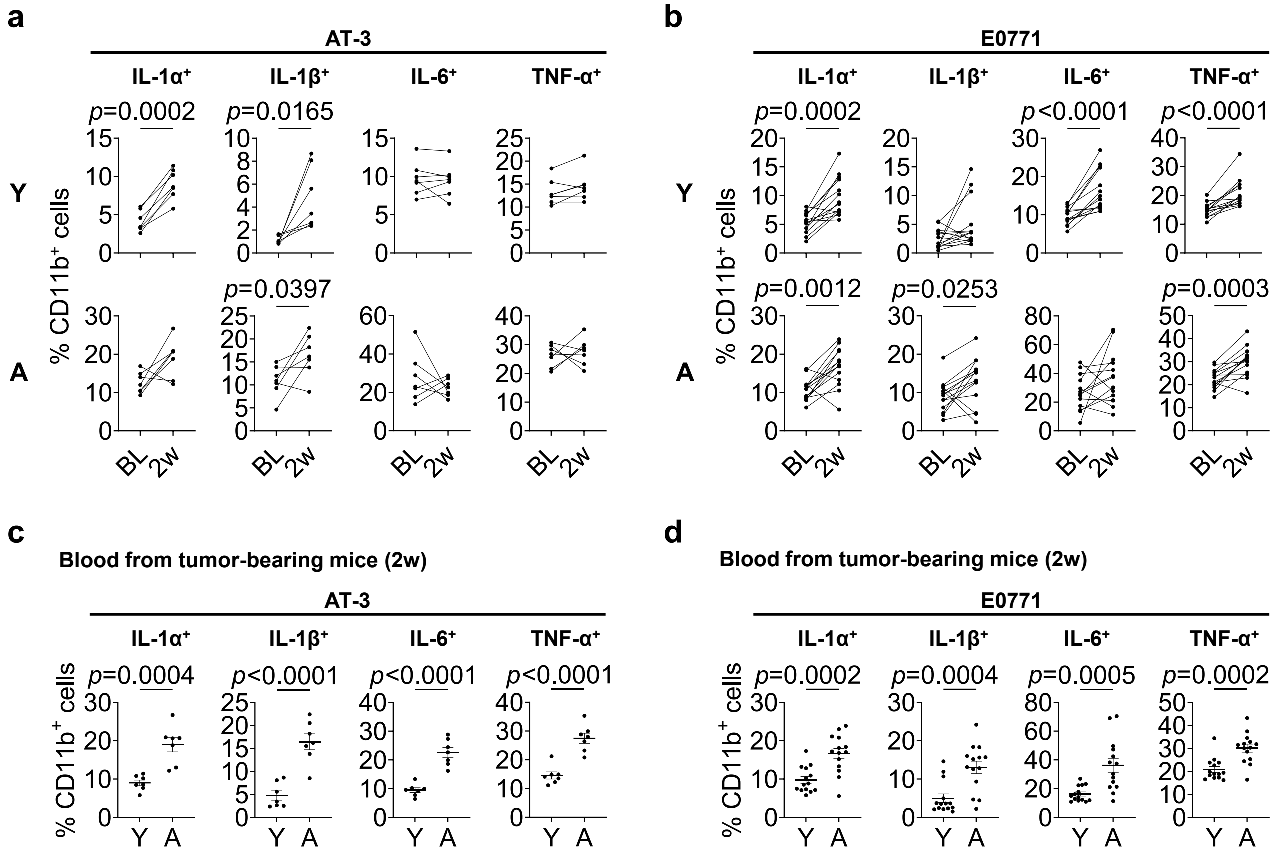

Supplementary Fig. 4. Impact of tumor presence and aging on circulating pro-inflammatory myeloid cells, related to Figure 1.

a–**d** Frequencies of cytokine-producing cells among CD11b⁺ blood cells from young (Y) and aged (A) female mice were evaluated at baseline (BL) and 2 weeks after tumor implantation (2w) (**a**, **c**, AT-3, n = 7 per group; **b**, **d**, E0771, n = 14 per group). Changes of cytokine-producing cells were compared before and after establishment of tumors each mouse (**a**, **b**). Frequency of cytokine-producing cells among CD11b⁺ blood cells between young (Y) and aged (A) mice at 2 weeks after tumor implantation was assessed (**c**, **d**).

Statistical significance was assessed using two-tailed paired (**a**, **b**) and unpaired (**c**, **d**) t-tests. Mean ± SEM. n values represent independent mice.

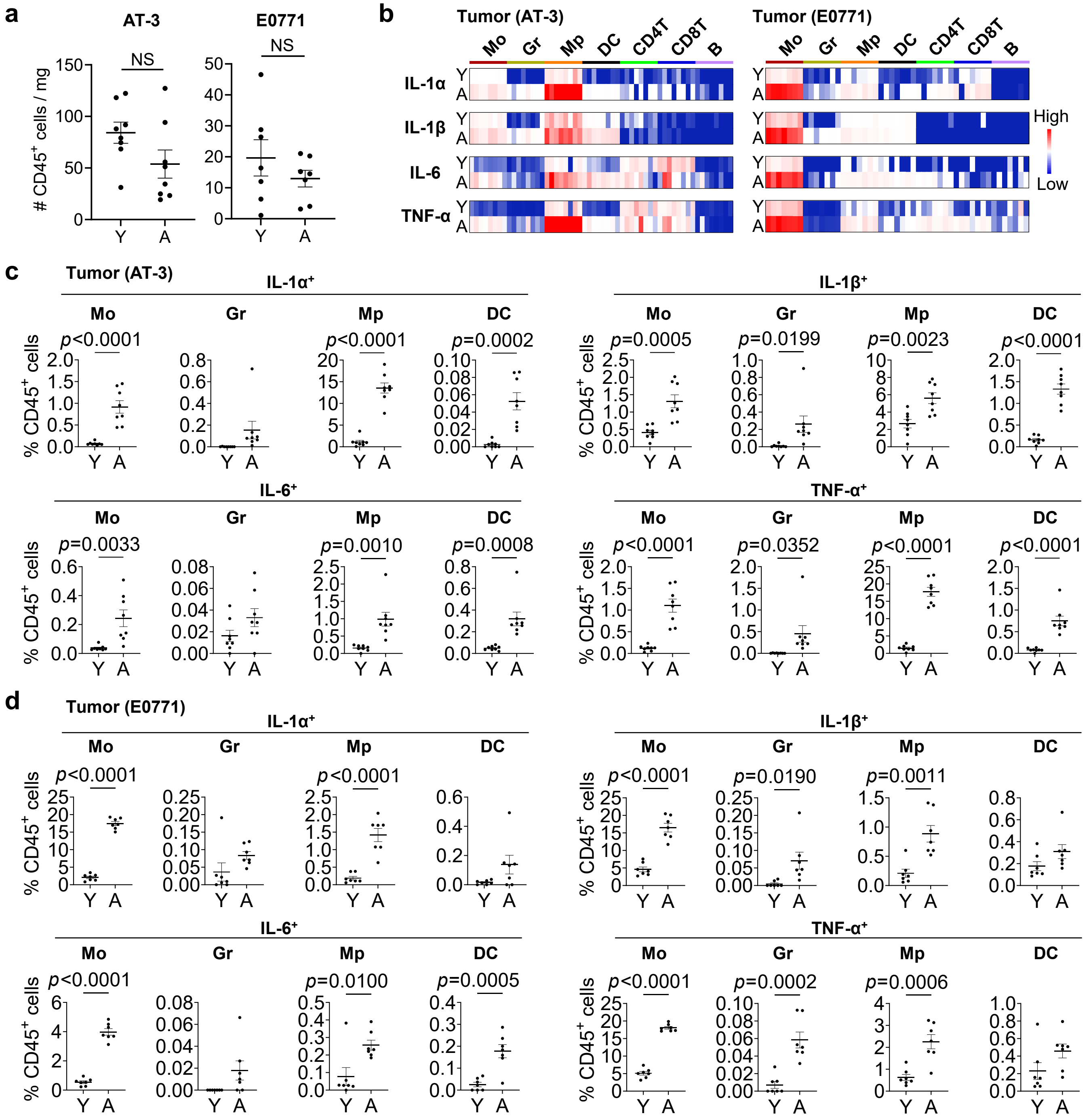

Supplementary Fig. 5. Monocytes and macrophages are the cytokine-producing predominant subsets in tumors, related to Figure 1.

a–d Flow cytometry analysis of AT-3 (n = 8 per group) and E0771 (n = 7 per group) tumor-infiltrating immune cells.

a Number of $CD45^+$ immune cells per mg of tumor tissue.

b Frequency of cytokine-producing cells within AT-3 (left) and E0771 (right) tumor-infiltrating $CD45^+$ cells from young (Y) and aged (A) mice shown as heatmaps.

c, **d** Frequency of each cytokine-producing myeloid subsets within AT-3 (**c**) and E0771 (**d**) tumor-infiltrating $CD45^+$ cells.

Statistical significance was assessed using two-tailed t-tests (**a**, **c**, **d**). Mean ± SEM. Mo, monocytes; Gr, granulocytes; Mp, macrophages; DC, dendritic cells; CD4T, $CD4^+$ T cells; CD8T, $CD8^+$ T cells; B, B cells. n values represent independent mice.

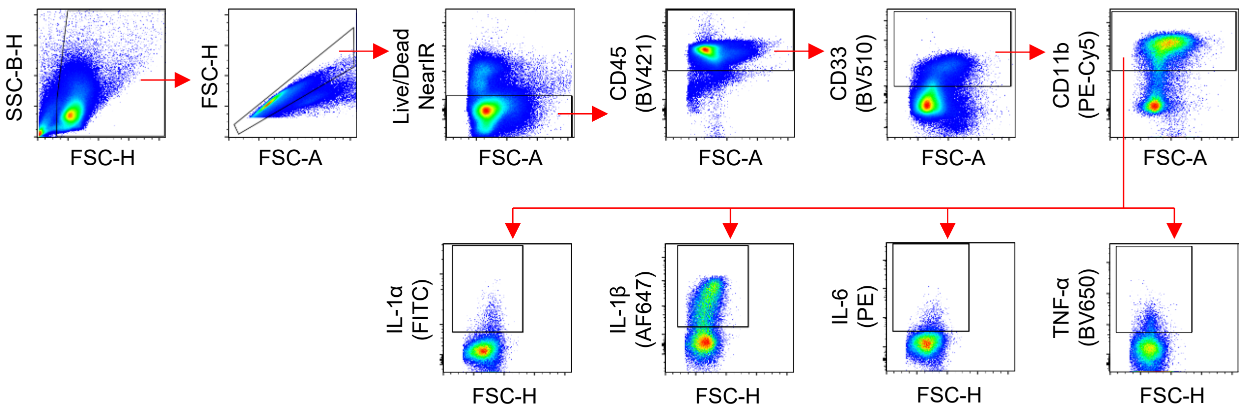

Supplementary Fig. 6. Gating strategy for cytokine-producing CD33^{+} CD11b^{+} myeloid cells in human PBMCs, related to Figure 2.
Cells were gated on singlets and live cells, followed by selection of CD45^{+}CD33^{+}CD11b^{+} myeloid cells. Cytokine-positive gates were defined using fluorescence minus one (FMO) controls.

Supplementary Fig. 7

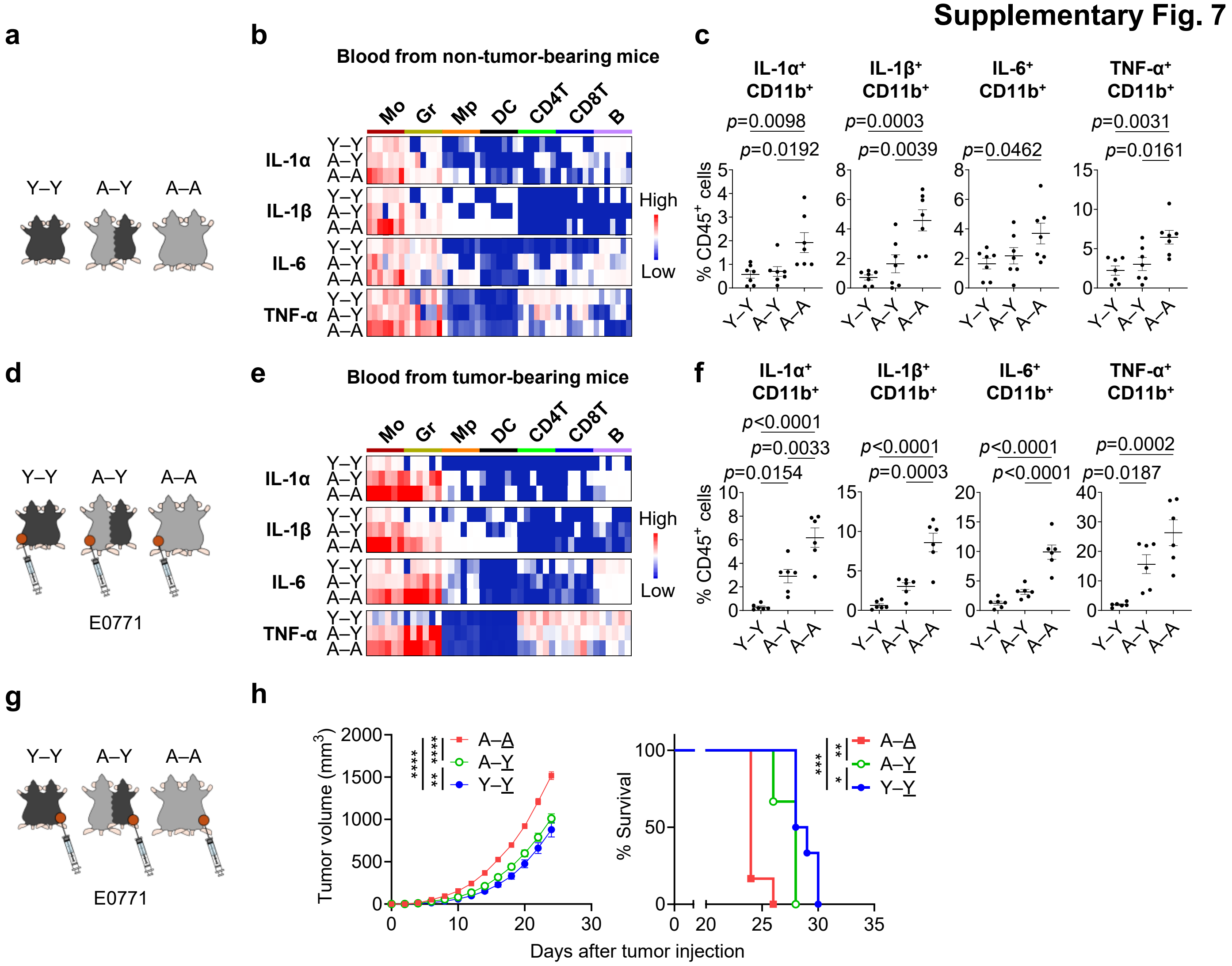

Supplementary Fig. 7. Exposure to a young systemic environment reduces pro-inflammatory myeloid cells, related to Figure 3.

a Experimental schematic of parabiosis.

b, **c** Frequency of cytokine-producing CD11b$^+$ cells within CD45$^+$ blood cells from non-tumor-bearing parabiotic mice (n = 7 pairs) shown as a heatmap (**b**) and quantified in data panels (**c**).

d Experimental setup for tumor-bearing parabiosis.

e, **f** Frequency of cytokine-producing CD11b$^+$ cells within CD45$^+$ blood cells from E0771 tumor-bearing parabiotic mice (**d**) (n = 6 pairs) shown as a heatmap (**e**) and quantified in data panels (**f**).

g Experimental setup for tumor-bearing parabiosis.

h Tumor growth curves (mean, left) and survival curves (right) of E0771 tumor-bearing parabiotic mice (**g**) (n = 6 pairs). Underlined labels indicate tumor-bearing parabionts. *$p < 0.05$; **$p < 0.01$, ***$p < 0.001$; ****$p < 0.0001$. Exact p values are provided in the Source Data. Statistical significance was assessed using two-tailed t-tests (**c**, **f**) and two-way ANOVA with Bonferroni's multiple comparisons test (tumor growth) and log-rank test (survival) (**h**). Mean ± SEM. Mo, monocytes; Gr, granulocytes; Mp, macrophages; DC, dendritic cells; CD4T, CD4$^+$ T cells; CD8T, CD8$^+$ T cells; B, B cells. n values represent independent pairs.

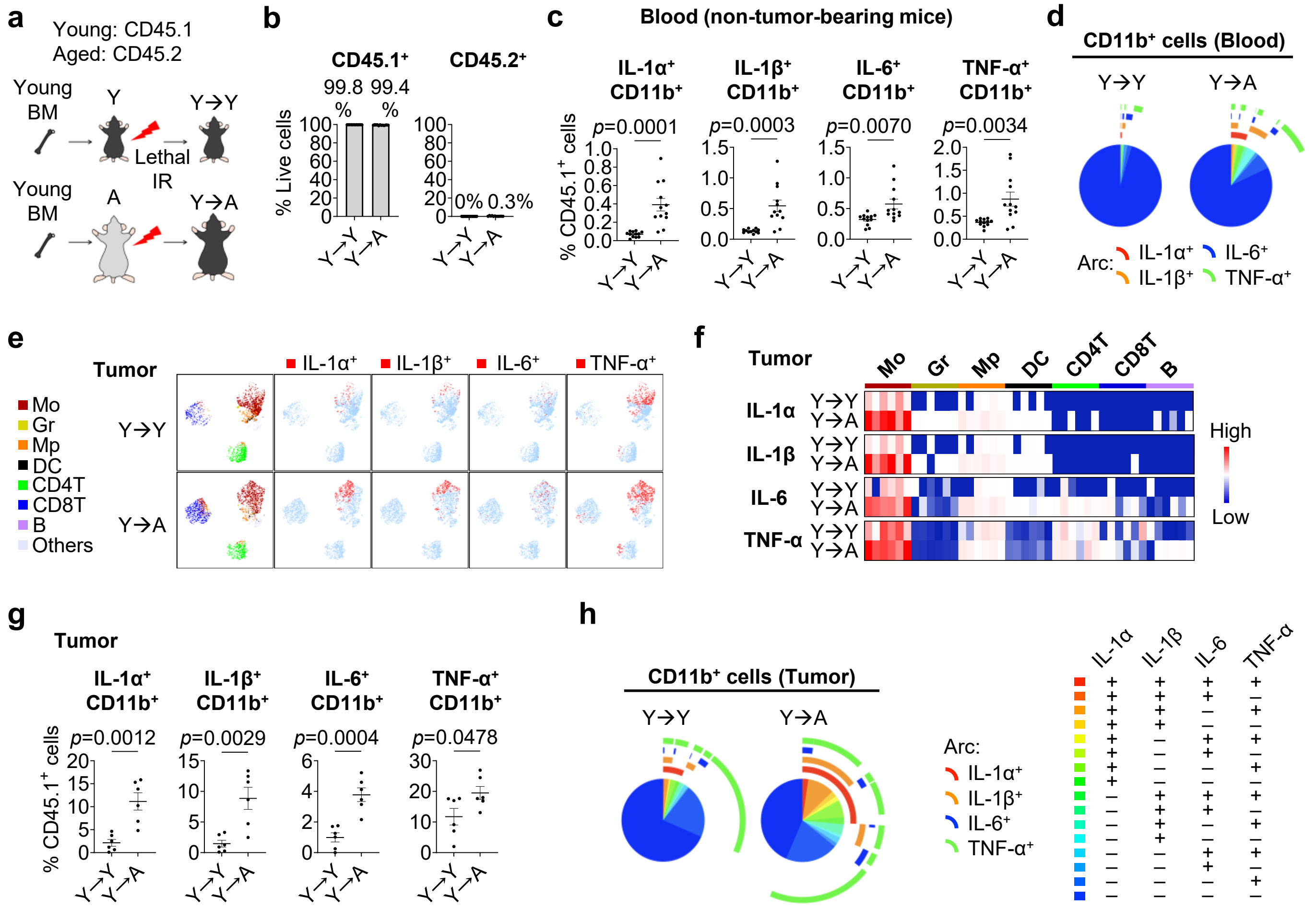

Supplementary Fig. 8. Young bone marrow-derived myeloid cells exhibit increased inflammatory activation in peripheral blood and tumors in aged hosts, related to Figure 4.

a–**d** Young (Y; CD45.1) or aged (A; CD45.2) recipient mice were irradiated (IR) and reconstituted with bone marrow from young or aged donors to generate Y→Y and Y→A chimeras (*n* = 12 per group).

a Experimental schematic of bone marrow chimera generation.

b Donor chimerism showing the proportions of CD45.1^{+} and CD45.2^{+} cells among total live blood cells.

c, **d** Frequency of cytokine-producing CD45.1^{+} blood cells in chimeric mice, shown as quantified data (**c**), with representative Boolean analysis (**d**).

e–**h** Flow cytometry analysis of CD45.1^{+} young bone marrow-derived cells in E0771 tumors from chimeric mice.

e Representative UMAP image of CD45.1^{+} tumor-infiltrating cells from chimeric mice.

f–**h** Frequency of cytokine-producing cells within CD45.1^{+} tumor-infiltrating cells from chimeric mice (*n* = 6 per group), shown as a heatmap (**f**) and quantified in data panels (**g**), with representative Boolean analysis (**h**).

Statistical significance was assessed using two-tailed *t*-tests (**c**, **g**). Mean ± SEM. Mo, monocytes; Gr, granulocytes; Mp, macrophages; DC, dendritic cells; CD4T, CD4^{+} T cells; CD8T, CD8^{+} T cells; B, B cells. *n* values represent independent mice.

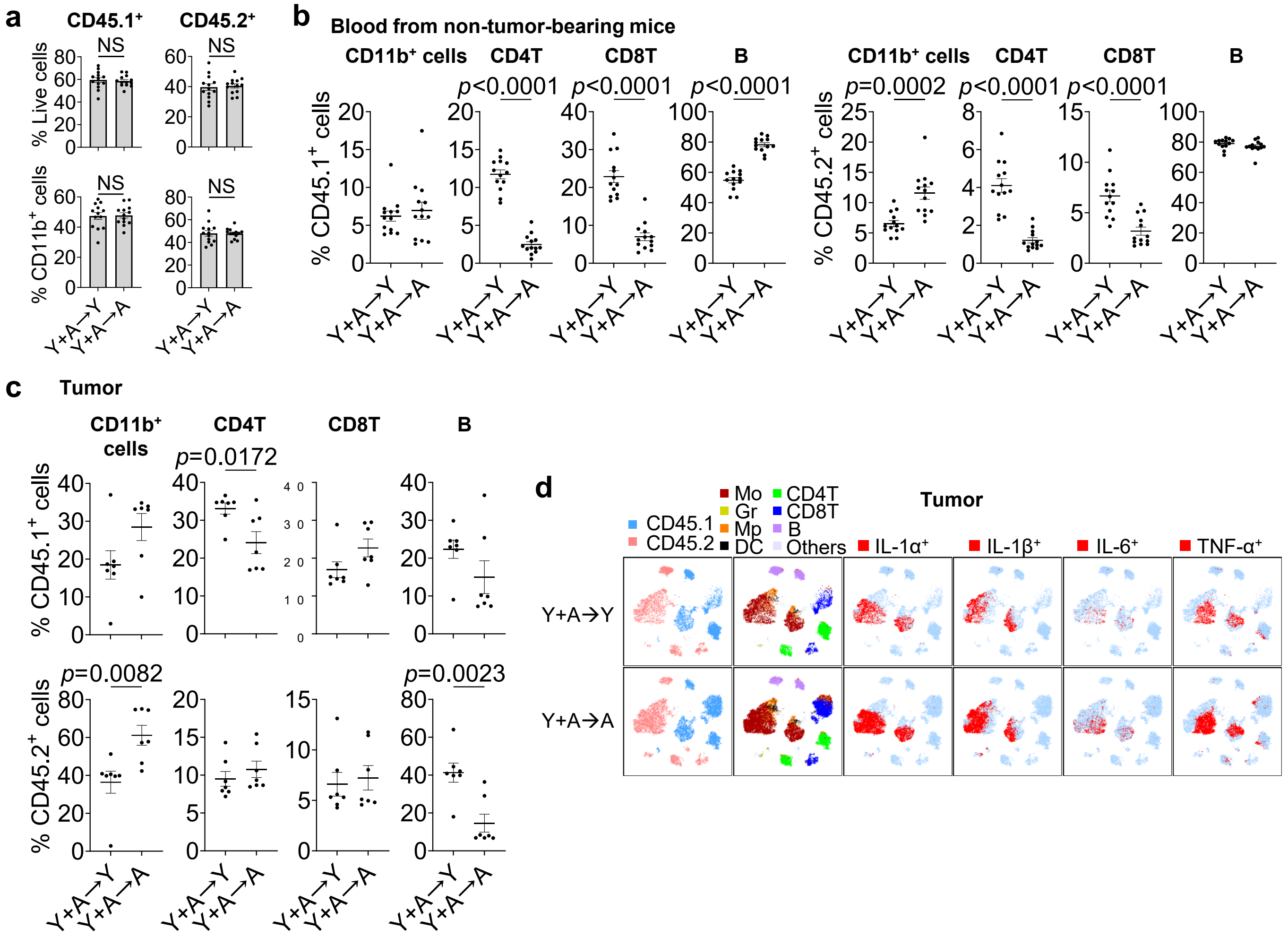

Supplementary Fig. 9. Immune composition in heterochronic mixed bone marrow chimeras, related to Figure 5.

a–d Flow cytometry analysis of circulating CD45.1$^+$ young and CD45.2$^+$ aged bone marrow-derived cells in heterochronic mixed bone marrow chimeras described in Fig. 5a (n = 13 per group).

a Donor chimerism showing proportions of CD45.1$^+$ and CD45.2$^+$ blood cells in mixed chimeric mice.

b Frequency of immune cell subsets within CD45.1 or CD45.2 blood cells from the non-tumor-bearing mixed chimeric mice, shown as quantified data.

c–d Flow cytometry analysis of CD45.1$^+$ young bone marrow-derived cells in E0771 tumors from heterochronic mixed bone marrow chimeric mice (n = 7 per group).

c Frequency of immune cell subsets within CD45.1 or CD45.2 tumor-infiltrating cells, shown as quantified data.

d Representative UMAP image of tumor-infiltrating CD45.1$^+$ and CD45.2$^+$ cells.

Statistical significance was assessed using two-tailed t-tests (**a–c**). Mean ± SEM. Mo, monocytes; Gr, granulocytes; Mp, macrophages; DC, dendritic cells; CD4T, CD4$^+$ T cells; CD8T, CD8$^+$ T cells; B, B cells. NS, not significant. n values represent independent mice.

Supplementary Fig. 10

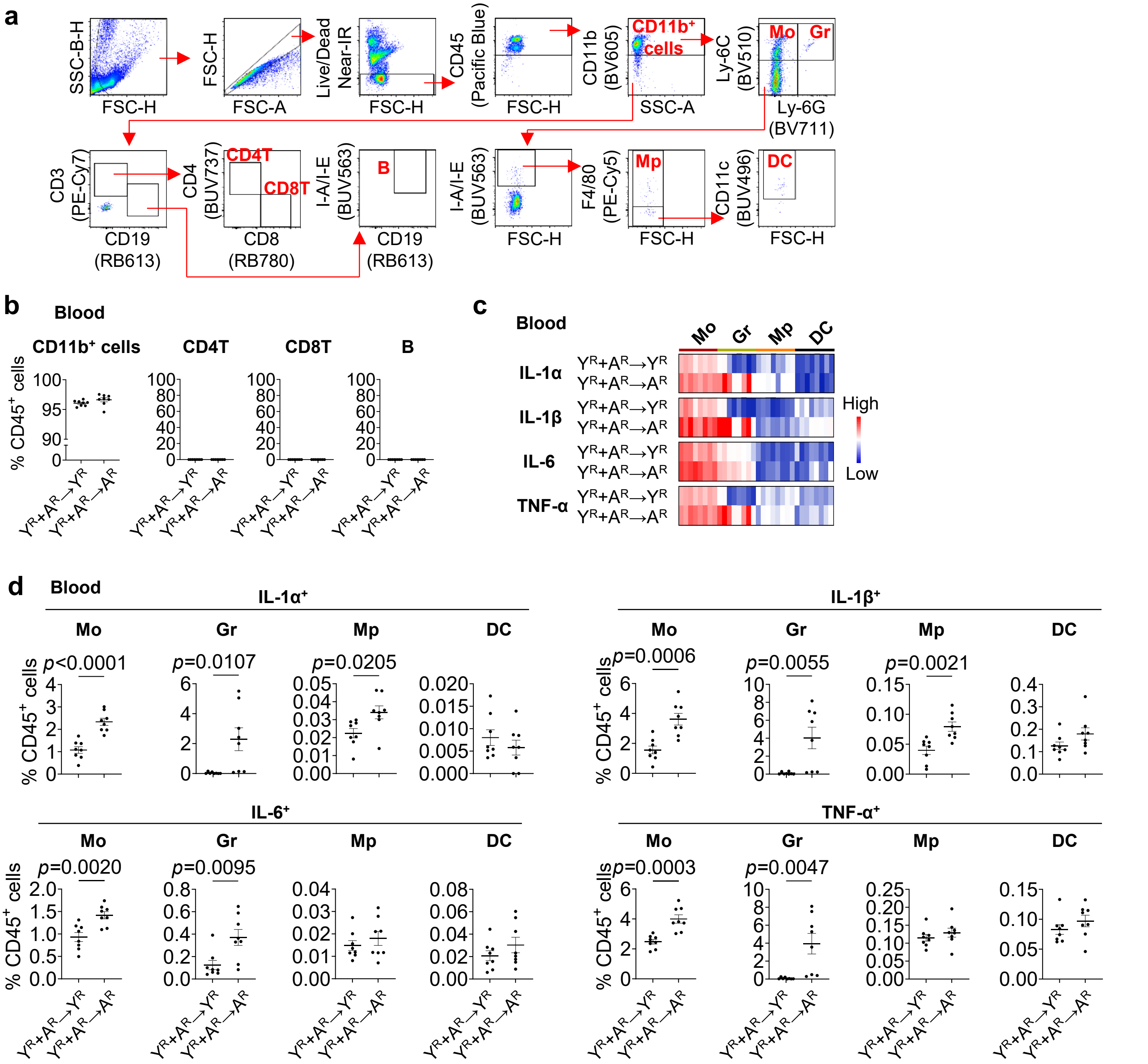

Supplementary Fig. 10. Host age dictates myeloid cell inflammatory activation in heterochronic mixed bone marrow chimeras independent of adaptive immunity, related to Figure 5.

a–**d** Flow cytometry analysis of circulating $CD45^+$ young and aged bone marrow-derived cells in Rag2-deficient ($Rag2^{-/-}$) heterochronic mixed bone marrow chimeras described in Fig. 5h ($n = 8$ per group).

a Gating strategy for identifying monocytes (Mo), granulocytes (Gr), macrophages (Mp), dendritic cells (DC), $CD4^+$ T cells (CD4T), $CD8^+$ T cells (CD8T), B cells (B) and the cytokine-producing $CD11b^+$ cells.

b Frequency of blood immune cell subsets in $Rag2^{-/-}$ mixed chimeric mice without tumors, shown as quantified data panels.

c, **d** Frequency of cytokine-producing cells within CD45 lineage-positive blood cells in mixed chimeric mice without tumors, shown as a heatmap (**c**) and quantified data panels (**d**).

Statistical significance was assessed using two-tailed *t*-tests (**b**, **d**). Mean ± SEM. *n* values represent independent mice.

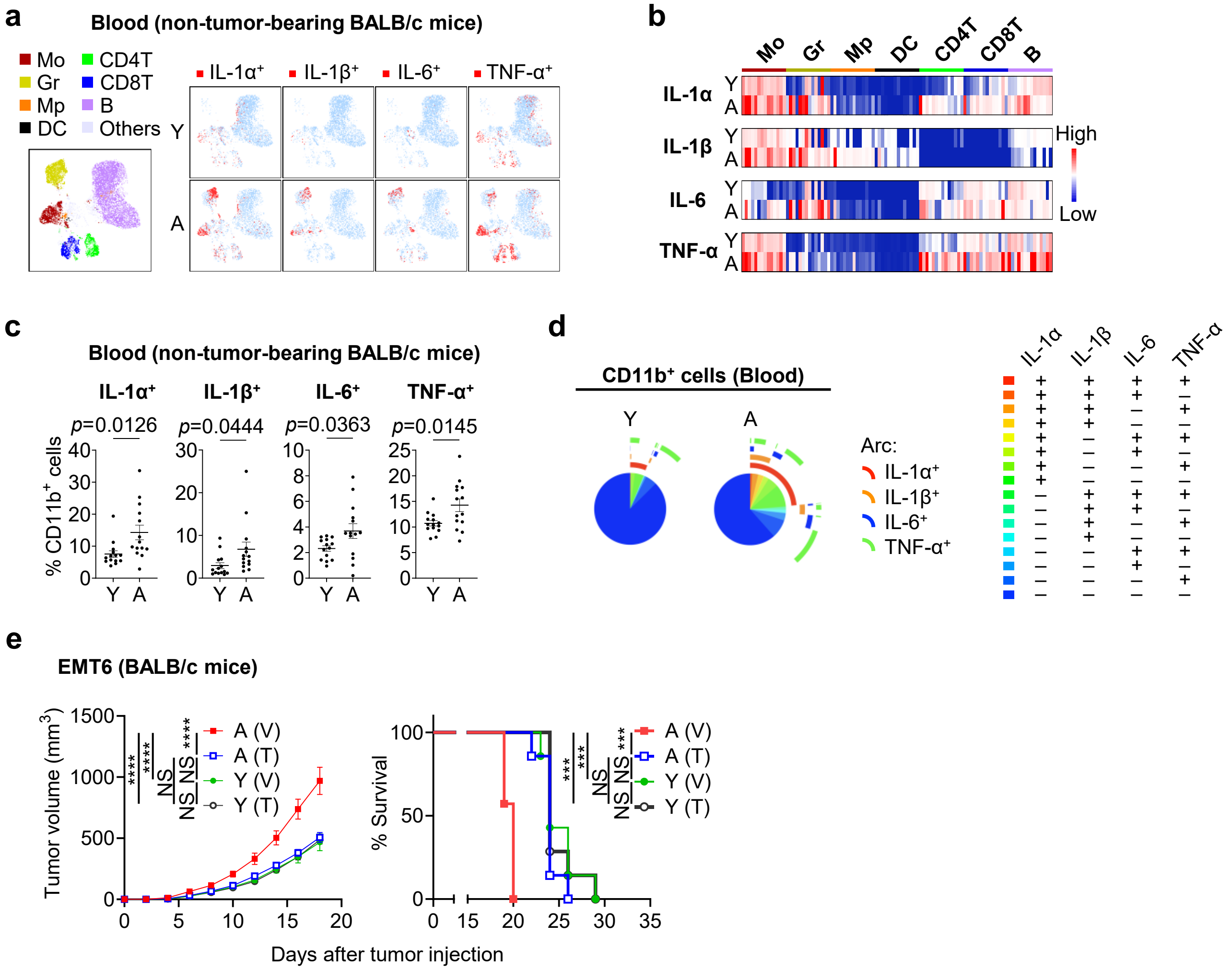

Supplementary Fig. 11. Pro-inflammatory cytokine–producing myeloid cells, tumor progression and response to thymulin in young and aged BALB/c mice, related to Figure 7.
a–d Flow cytometry analysis of circulating $CD45^+$ cells in young and aged BALB/c female mice without tumors ($n = 14$ per group).
a Representative UMAP.
b–d Frequencies of cytokine-positive cells among $CD11b^+$ blood cells, shown as a heatmap (**b**), individual data plots (**c**), and representative Boolean analysis (**d**).
e Tumor growth (mean) and survival of EMT6 tumor-bearing BALB/c mice treated with vehicle or thymulin ($n = 7$ per group).
Two-tailed t-test (**c**). ***$p < 0.001$; ****$p < 0.0001$ by two-way ANOVA with Bonferroni's multiple comparisons test (tumor growth) and log-rank test (survival) (**e**); NS, not significant. Exact p values are provided in the Source Data. Mean ± SEM. Mo, monocytes; Gr, granulocytes; Mp, macrophages; DC, dendritic cells; CD4T, $CD4^+$ T cells; CD8T, $CD8^+$ T cells; B, B cells; V, vehicle; T, thymulin. n values represent independent mice.

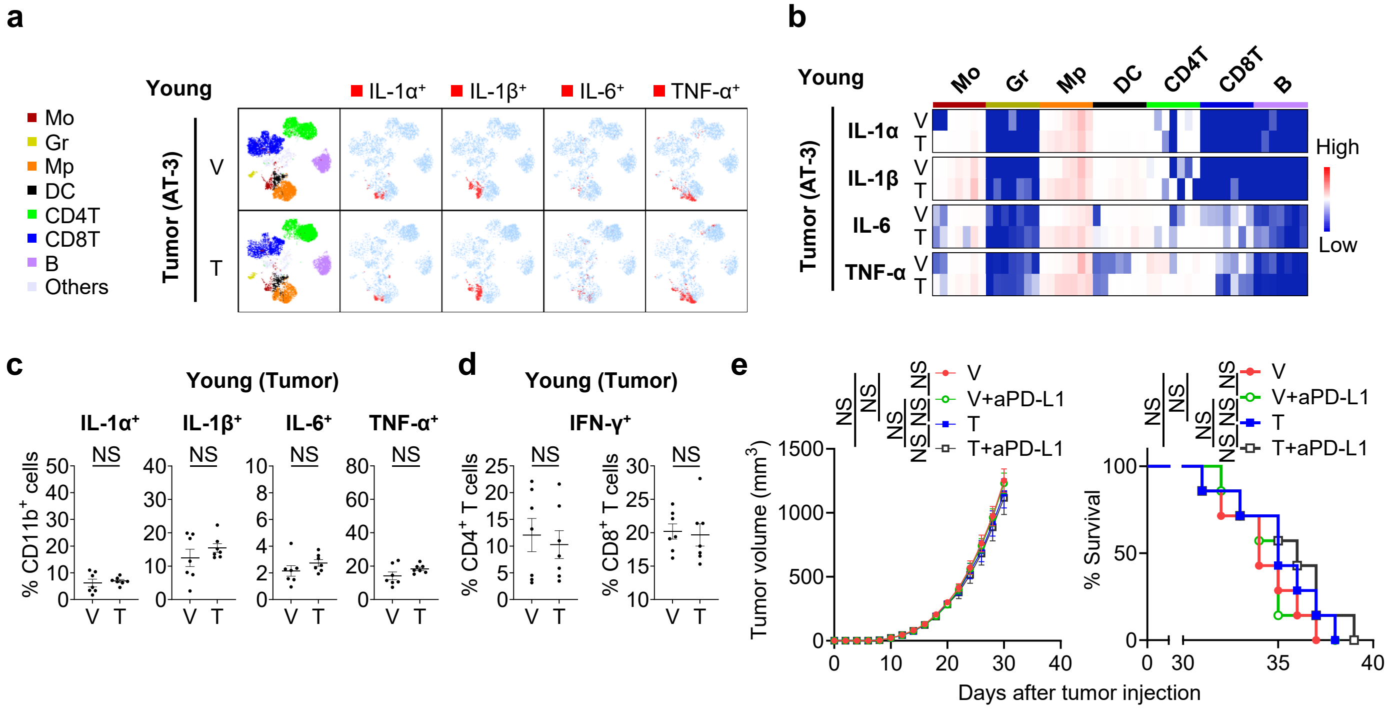

Supplementary Fig. 12. Thymulin effects are limited in young mice, related to Figure 7.
a–**d** Flow cytometry analysis of AT-3 tumor-infiltrating $CD45^+$ cells from young mice treated with vehicle or thymulin (*n* = 7 per group).
a Representative UMAP image.
b, **c** Frequency of pro-inflammatory cytokine (IL-1α, IL-1β, IL-6, and TNF-α)-producing cells among $CD45^+$ (**b**) and $CD11b^+$ (**c**) tumor-infiltrating cells in young mice treated with vehicle or thymulin, shown as a heatmap (**b**) and quantified data panels (**c**).
d Frequency of IFN-γ-producing $CD4^+$ and $CD8^+$ T cells within AT-3 tumor-infiltrating $CD45^+$ cells from young mice treated with vehicle or thymulin.
e Tumor growth curves (mean, left) and survival curves (right) of AT-3 tumor-bearing mice treated with thymulin and/or anti-PD-L1 antibody (*n* = 7 per group).
Statistical significance was assessed using two-tailed *t*-tests (**c**, **d**), two-way ANOVA with Bonferroni's multiple comparisons test (tumor growth) and log-rank test (survival) (**e**); NS, not significant. Mean ± SEM. V: vehicle; T: thymulin; Mo, monocytes; Gr, granulocytes; Mp, macrophages; DC, dendritic cells; CD4T, $CD4^+$ T cells; CD8T, $CD8^+$ T cells; B, B cells. *n* values represent independent mice.

Supplementary Table 1. List of antibodies

Antibodies	Source	Catalog Number	Dilution
Anti-human CD11b clone ICRF44 PE-Cy5	BioLegend	Cat# 301308	1:17
Anti-human CD33 clone P67.6 BV510	BioLegend	Cat# 366610	1:10
Anti-human CD45 clone HI30 BV421	BD Biosciences	Cat# 563879	1:25
Anti-human IL-1α clone 364/3B3-14 FITC	Thermo Fisher scientific	Cat# 11-7118-82	1:10
Anti-human IL-1β clone CRM56 Alexa Fluor 647	Thermo Fisher scientific	Cat# 51-7018-42	1:10
Anti-human IL-6 clone MQ2-13A5 PE	BioLegend	Cat# 501107	1:10
Anti-human TNF-α clone MAb11 BV650	BD Biosciences	Cat# 563418	1:10
Anti-mouse CD11b clone M1/70 BV605	Thermo Fisher scientific	Cat# 406-0112-82	1:100
Anti-mouse CD11c clone N418 BUV496	BD Biosciences	Cat# 750450	1:100
Anti-mouse CD19 clone 1D3 RB613	BD Biosciences	Cat# 571233	1:100
Anti-mouse CD3ε clone 145-2C11 PE-Cy7	BioLegend	Cat# 100320	1:100
Anti-mouse CD4 clone GK1.5 BUV737	BD Biosciences	Cat# 612761	1:100
Anti-mouse CD45 clone 30-F11 Pacific Blue	BioLegend	Cat# 103126	1:100
Anti-mouse CD45 clone 30-F11 PE-Cy5	BioLegend	Cat# 103110	1:100
Anti-mouse CD45.1 clone A20 BV750	BD Biosciences	Cat# 747314	1:100
Anti-mouse CD45.2 clone 104 Pacific Blue	BioLegend	Cat# 109820	1:100
Anti-mouse CD8 alpha clone 53-6.7 BUV395	BD Biosciences	Cat# 563786	1:100
Anti-mouse CD8 alpha clone 53-6.7 RB780	BD Biosciences	Cat# 568692	1:100
Anti-mouse F4/80 clone BM8 BV650	BioLegend	Cat# 123149	1:100
Anti-mouse F4/80 clone BM8 PE-Cy5	BioLegend	Cat# 123112	1:100
Anti-mouse I-A/I-E clone M5/114.15.2 BUV563	BD Biosciences	Cat# 748846	1:100
Anti-mouse IFN-γ XMG1.2 Alexa Fluor 700	BioLegend	Cat# 505824	1:100
Anti-mouse IL-1α clone ALF-161 PE	Thermo Fisher scientific	Cat# 12-7011-82	1:100
Anti-mouse IL-1β clone NJTEN3 FITC	Thermo Fisher scientific	Cat# 11-7114-82	1:200
Anti-mouse IL-6 clone MP5-20F3 APC	BioLegend	Cat# 504508	1:50
Anti-mouse Ly-6C clone HK1.4 BV510	BioLegend	Cat# 128033	1:200
Anti-mouse Ly-6G clone 1A8 BV711	BioLegend	Cat# 127643	1:100
Anti-mouse TNF-α clone MP6-XT22 RB705	BD Biosciences	Cat# 570733	1:100
LIVE/DEAD Fixable Near-IR Dead Cell Stain Kit	Thermo Fisher scientific	L34976	1:200

INTERNAL
