# Supplementary material for: Thymulin restrains age-associated myeloid inflammation and enhances cancer immunotherapy
Source: Nat Commun. 2026 Jul 21;17:6534. doi: 10.1038/s41467-026-75383-0 (PMC13389034; doi:10.1038/s41467-026-75383-0)
Supplement: Supplementary file 2 — Description of Additional Supplementary Files [file 41467_2026_75383_MOESM2_ESM.pdf]

Descriptions of Additional Supplementary Files

Supplementary Dataset 1: Gene expression patterns significantly associated with young (n = 8) relative to aged (n = 8) patients were determined by comparing myeloid cells from the pooled, TNBC, HER2+ and ER+ tumor microenvironments (GSE176078). Differential expression was implemented in Seurat via the MAST method implemented in the FindMarkers function. Each row represents a single gene, and columns provide the raw p-value (p_val) of observed differences, the average log fold change (avg_log2FC), the fraction of cells with positive detection of the given Fumito Ito, MD, PhD, FACS Professor of Surgery & Immunology and Immune Therapeutics Keck School of Medicine, University of Southern California Co-Leader of the Translational and Clinical Sciences Research Director, Flow Cytometry and Immune Monitoring Core Norris Comprehensive Cancer Center 1450 Biggy St, NRT 3505 Los Angeles, California 90033 Office: 323-442-8140 Fumito.Ito@med.usc.edu gene in the aged myeloid population (pct.1) versus the young myeloid population (pct.2), bonferroni adjusted P value (p_val_adj), and its significance (significant) within aged myeloid cells relative to young myeloid cells using the following thresholds (‘Upregulated’ = p_val_adj < 0.05 and avg_log2FC > 0.1; ‘Downregulated’ = p_val_adj < 0.05 and avg_log2FC < 0.1; ‘NS’ = p_val_adj > 0.05).

Supplementary Dataset 2: VISION-based single-cell pathway activity analysis was performed on myeloid cells from young and aged patients using curated gene signatures from the Molecular Signature Database (MSigDB) including GO-Biological Processes, KEGG, REACTOME, BIOCARTA, PID, and HALLMARK collections. Per-cell pathway activity scores were computed from SCTransform-normalized expression data, averaged for each patient, and subsequently znormalized per pathway to compare relative pathway activity across patients.

Supplementary Dataset 3: List of 163 molecules associated with “Aging,” were identified using the QIAGEN Ingenuity Pathway Analysis (IPA) database (“Diseases and Functions” category). Molecules were retrieved using “Aging” as the query term and used for subsequent analyses.
