# Supplementary material for: Thymulin restrains age-associated myeloid inflammation and enhances cancer immunotherapy
Source: Nat Commun. 2026 Jul 21;17:6534. doi: 10.1038/s41467-026-75383-0 (PMC13389034; doi:10.1038/s41467-026-75383-0)
Supplement: Supplementary file 6 — Reporting Summary [file 41467_2026_75383_MOESM6_ESM.pdf]

nature portfolio

Corresponding author(s): Fumito Ito

Last updated by author(s): 2026/5/28

Reporting Summary

Nature Portfolio wishes to improve the reproducibility of the work that we publish. This form provides structure for consistency and transparency in reporting. For further information on Nature Portfolio policies, see our Editorial Policies and the Editorial Policy Checklist.

Statistics

For all statistical analyses, confirm that the following items are present in the figure legend, table legend, main text, or Methods section.

n/a	Confirmed	
☐	☒	The exact sample size (*n*) for each experimental group/condition, given as a discrete number and unit of measurement
☐	☒	A statement on whether measurements were taken from distinct samples or whether the same sample was measured repeatedly
☐	☒	The statistical test(s) used AND whether they are one- or two-sided<br>*Only common tests should be described solely by name; describe more complex techniques in the Methods section.*
☒	☐	A description of all covariates tested
☐	☒	A description of any assumptions or corrections, such as tests of normality and adjustment for multiple comparisons
☐	☒	A full description of the statistical parameters including central tendency (e.g. means) or other basic estimates (e.g. regression coefficient) AND variation (e.g. standard deviation) or associated estimates of uncertainty (e.g. confidence intervals)
☐	☒	For null hypothesis testing, the test statistic (e.g. *F*, *t*, *r*) with confidence intervals, effect sizes, degrees of freedom and *P* value noted<br>*Give P values as exact values whenever suitable.*
☒	☐	For Bayesian analysis, information on the choice of priors and Markov chain Monte Carlo settings
☒	☐	For hierarchical and complex designs, identification of the appropriate level for tests and full reporting of outcomes
☐	☒	Estimates of effect sizes (e.g. Cohen's *d*, Pearson's *r*), indicating how they were calculated

Our web collection on statistics for biologists contains articles on many of the points above.

Software and code

Policy information about availability of computer code

Data collection	Aurora (flow cytometry), CLARIOstar Plus (microplate reader), iBright 1500 (immunoblot imager)
Data analysis	Flow cytometric data were analyzed using FlowJo (v10.10.0), SPICE (v6.1), and Origin (2024b, v10.1.5.132).<br>scRNA-seq data were analyzed using R (v4.5.2) with the packages DoubletFinder (v2.0.6), Seurat (v5.4.0), and VISION (v3.0.2).<br>Statistical analyses were performed using GraphPad Prism (v10.5.0).

For manuscripts utilizing custom algorithms or software that are central to the research but not yet described in published literature, software must be made available to editors and reviewers. We strongly encourage code deposition in a community repository (e.g. GitHub). See the Nature Portfolio guidelines for submitting code & software for further information.

Data

Policy information about availability of data

All manuscripts must include a data availability statement. This statement should provide the following information, where applicable:

- Accession codes, unique identifiers, or web links for publicly available datasets
- A description of any restrictions on data availability
- For clinical datasets or third party data, please ensure that the statement adheres to our policy

The source data underlying the graphs in Figs. 1–7 and Supplementary Figs. 2–5 and 7–12 have been provided as a Source Data file. The datasets analyzed in Fig. 2 are publicly available in the Gene Expression Omnibus (GEO) repository under accession number GSE176078. The QIAGEN Ingenuity Pathway Analysis (IPA)

database was used to identify candidate factors associated with aging and inflammation in Fig. 6. All data generated or analyzed in this study are available from the corresponding author upon reasonable request.

Research involving human participants, their data, or biological material

Policy information about studies with human participants or human data. See also policy information about sex, gender (identity/presentation), and sexual orientation and race, ethnicity and racism.

Reporting on sex and gender	Both male and female healthy adult volunteers were included as donors for peripheral blood mononuclear cell (PBMC) collection. Donor sex was self-reported at the time of enrollment. PBMCs were derived from venous blood and used in experiments without stratification by sex. The study did not perform sex-specific analyses due to sample size considerations.
Reporting on race, ethnicity, or other socially relevant groupings	Information on race, ethnicity, or other socially constructed groupings was not collected for the healthy volunteer PBMC donors in this study. Participants were enrolled based on defined inclusion/exclusion criteria irrespective of race or ethnicity. No analyses based on race or ethnicity were conducted, and these variables were not used as proxies for other social determinants.
Population characteristics	The ages of the patients included in this study were as follows: Fig. 2a (21–87 years), Fig. 2b (young: 21–33 years; aged: 60–87 years), and Fig. 6c (young: 21–28 years; aged: 52–86 years).
Recruitment	Written informed consent was obtained from 93 healthy volunteers aged between 21 and 87 with no personal history of cancer for the collection, storage and analysis of blood samples.
Ethics oversight	Written informed consent was obtained under the Institutional Review Board of the University of Southern California (approval number: HS-22-00354) in accordance with the Declaration of Helsinki.

Note that full information on the approval of the study protocol must also be provided in the manuscript.

Field-specific reporting

Please select the one below that is the best fit for your research. If you are not sure, read the appropriate sections before making your selection.

☒ Life sciences ☐ Behavioural & social sciences ☐ Ecological, evolutionary & environmental sciences

For a reference copy of the document with all sections, see nature.com/documents/nr-reporting-summary-flat.pdf

Life sciences study design

All studies must disclose on these points even when the disclosure is negative.

Sample size	Sample sizes were determined based on our and other investigators experience with the respective cell lines used (e.g.: Oba et al. 2020 (PMID: 33110069), Yamauchi et al. 2020 (PMID: 32255766), Oba et al. 2020 (PMID: 32848036), Mohammadpour et al. 2019 (PMID: 31566578), Twum et al. 2019 (PMID: 30728331), Juneja et al. 2017 (PMID: 28302645), Saito et al. 2016 (PMID: 27197199), Ngiow et al 2015 (PMID: 26208901)). No statistical methods were used as we observed many statistically significant effects in the data with the above methods of sample size selection without a priori sample size calculations.
Data exclusions	No data were excluded from the analyses.
Replication	Data in Fig. 1a–g, Fig. 3 a–h, Fig. 4 a–e, Fig. 5 a–d, Fig. 6 d–k, Fig. 7a, b, and Supplementary Figs. 2, 4a-d, 5a-d, 7a-f, 8a-h, 9a, b, 12a-d are representative of two or three independent experiments. No technical replicates were treated as independent samples in statistical analyses.
Randomization	Stratified randomization was used based on tumor size to ensure equal distribution of tumor sizes within each group.
Blinding	Blinding was not possible, since all groups were treated differently and injected repeatedly, so it was necessary for the investigators to know which animals belong to which group.

Reporting for specific materials, systems and methods

We require information from authors about some types of materials, experimental systems and methods used in many studies. Here, indicate whether each material, system or method listed is relevant to your study. If you are not sure if a list item applies to your research, read the appropriate section before selecting a response.

Materials & experimental systems

n/a	Involved in the study	
	☒	Antibodies
	☒	Eukaryotic cell lines
☒		Palaeontology and archaeology
	☒	Animals and other organisms
☒		Clinical data
☒		Dual use research of concern
☒		Plants

Methods

n/a	Involved in the study	
☒		ChIP-seq
	☒	Flow cytometry
☒		MRI-based neuroimaging

Antibodies

Antibodies used

Flow cytometry:
Antibodies, Source, Catalog Number, Dilution
Anti-human CD11b clone ICRF44 PE-Cy5, BioLegend, Cat# 301308, 1:17
Anti-human CD33 clone P67.6 BV510, BioLegend, Cat# 366610, 1:10
Anti-human CD45 clone HI30 BV421, BD Biosciences, Cat# 563879, 1:25
Anti-human IL-1α clone 364/3B3-14 FITC, Thermo Fisher scientific, Cat# 11-7118-82, 1:10
Anti-human IL-1β clone CRM56 Alexa Fluor 647, Thermo Fisher scientific, Cat# 51-7018-42, 1:10
Anti-human IL-6 clone MQ2-13A5 PE, BioLegend, Cat# 501107, 1:10
Anti-human TNF-α clone MAb11 BV650, BD Biosciences, Cat# 563418, 1:10
Anti-mouse CD11b clone M1/70 BV605, Thermo Fisher scientific, Cat# 406-0112-82, 1:100
Anti-mouse CD11c clone N418 BUV496, BD Biosciences, Cat# 750450, 1:100
Anti-mouse CD19 clone 1D3 RB613, BD Biosciences, Cat# 571233, 1:100
Anti-mouse CD3ε clone 145-2C11 PE-Cy7, BioLegend, Cat# 100320, 1:100
Anti-mouse CD4 clone GK1.5 BUV737, BD Biosciences, Cat# 612761, 1:100
Anti-mouse CD45 clone 30-F11 Pacific Blue, BioLegend, Cat# 103126, 1:100
Anti-mouse CD45 clone 30-F11 PE-Cy5, BioLegend, Cat# 103110, 1:100
Anti-mouse CD45.1 clone A20 BV750, BD Biosciences, Cat# 747314, 1:100
Anti-mouse CD45.2 clone 104 Pacific Blue, BioLegend, Cat# 109820, 1:100
Anti-mouse CD8 alpha clone 53-6.7 BUV395, BD Biosciences, Cat# 563786, 1:100
Anti-mouse CD8 alpha clone 53-6.7 RB780, BD Biosciences, Cat# 568692, 1:100
Anti-mouse F4/80 clone BM8 BV650, BioLegend, Cat# 123149, 1:100
Anti-mouse F4/80 clone BM8 PE-Cy5, BioLegend, Cat# 123112, 1:100
Anti-mouse I-A/I-E clone M5/114.15.2 BUV563, BD Biosciences, Cat# 748846, 1:100
Anti-mouse IFN-γ XMG1.2 Alexa Fluor 700, BioLegend, Cat# 505824, 1:100
Anti-mouse IL-1α clone ALF-161 PE, Thermo Fisher scientific, Cat# 12-7011-82, 1:100
Anti-mouse IL-1β clone NJTEN3 FITC, Thermo Fisher scientific, Cat# 11-7114-82, 1:200
Anti-mouse IL-6 clone MP5-20F3 APC, BioLegend, Cat# 504508, 1:50
Anti-mouse Ly-6C clone HK1.4 BV510, BioLegend, Cat# 128033, 1:200
Anti-mouse Ly-6G clone 1A8 BV711, BioLegend, Cat# 127643, 1:100
Anti-mouse TNF-α clone MP6-XT22 RB705, BD Biosciences, Cat# 570733, 1:100
LIVE/DEAD Fixable Near-IR Dead Cell Stain Kit, Thermo Fisher scientific, L34976, 1:200

Immunoblot analysis:
Antibodies, Source, Catalog Number, Dilution
Anti-phospho-IκBα (Ser32/36) 5A5, Cell Signaling Technology, Cat# 9246, 1:1000
HRP anti-β-actin 2F1-1, BioLegend, Cat# 643807, 1:1000
HRP Goat anti-mouse IgG clone Poly4053, BioLegend, Cat# 405306, 1:2000

In vivo treatment:
InVivoMAb anti-mouse PD-L1 (B7-H1) 10F.9G2, # BE0101

Validation

All primary antibodies were validated by the manufacturers, and validation information is available on the manufacturers' websites.

Eukaryotic cell lines

Policy information about cell lines and Sex and Gender in Research

Cell line source(s)

The E0771 cell line was purchased from the CH3 BioSystems. B16-F10 (B16), RAW 264.7 and EMT6 cell lines were purchased from the American Type Culture Collection (ATCC). The AT-3 cell line was a gift from Dr. Scott Abrams (Roswell Park Comprehensive Cancer Center, Buffalo, NY).

Authentication

Cell lines obtained from external institutions were authenticated by morphology, phenotype and growth.

Mycoplasma contamination

Cells lines tested negative for mycoplasma contamination prior to sample generation. Samples were confirmed negative using MycoAlert (Lonza) Mycoplasma Detection Kit.

Commonly misidentified lines (See ICLAC register)	No commonly misidentified lines were used in this study.

Animals and other research organisms

Policy information about studies involving animals; ARRIVE guidelines recommended for reporting animal research, and Sex and Gender in Research

Laboratory animals	Female and male CD45.1 or CD45.2 C57BL/6 mice, as well as BALB/c mice, were obtained from Charles River Laboratories or The Jackson Laboratory. Rag2 knockout (Rag2–/–; B6.Cg-Rag2tm1.1Cgn/J) mice were obtained from The Jackson Laboratory, and were bred in-house. C57BL/6 mice aged 8–12 weeks and 65–75 weeks were used as the young and aged cohorts, respectively. For BALB/c mice used in this study, animals aged ≥65 weeks were defined as aged based on experimental availability. Mice were maintained under specific pathogen-free conditions with controlled temperature and humidity according to institutional guidelines.
Wild animals	N/A
Reporting on sex	Male mice data are shown in Supplementary Fig. 3. In other figures, female mice data are shown.
Field-collected samples	N/A
Ethics oversight	All animal experiments were performed in accordance with and approved by the Institutional Animal Care and Use Committee at University of Southern California.

Note that full information on the approval of the study protocol must also be provided in the manuscript.

Plants

Seed stocks	N/A
Novel plant genotypes	N/A
Authentication	N/A

Flow Cytometry

Plots

Confirm that:

- [x] The axis labels state the marker and fluorochrome used (e.g. CD4-FITC).
- [ ] The axis scales are clearly visible. Include numbers along axes only for bottom left plot of group (a 'group' is an analysis of identical markers).
- [x] All plots are contour plots with outliers or pseudocolor plots.
- [x] A numerical value for number of cells or percentage (with statistics) is provided.

Methodology

Sample preparation	Single-cell suspensions of mouse peripheral blood and tumors were prepared for flow cytometric analysis. Red blood cells in blood were lysed using ACK Lysis Buffer (Life Technologies). Tumor tissues were weighed, minced, filtered through 70-µm filters, and stored -80 °C in FBS (Hyclone, Waltham, MA) with 10% DMSO (Sigma) before analysis.
Instrument	Aurora (Cytek)
Software	Collection: SpectroFlo<br>Analysis: FlowJo 10.10.0, , SPICE v6.1, Origin 2024b 10.1.5.132, and GraphPad Prism 10.5.0.
Cell population abundance	N/A
Gating strategy	Gating strategy for identifying monocytes (Mo), granulocytes (Gr), macrophages (Mp), dendritic cells (DC), CD4+ T cells (CD4T), CD8+ T cells (CD8T), B cells (B) and the indicated cytokine-positive CD11b+ cells. Following gating to include singlets (FSC-H v.s. FSC-A) and live cells by Live/DEAD Fixable NearIR Dead Cell stain, cells were gated for CD45+ cells. Mo were identified as CD11b+ Ly6C+ Ly6G- cells among CD45+ live cells. Gr were identified as CD11b+ Ly6C+ Ly6G+ cells among CD45+ live cells. Mp were identified as CD11b+ Ly6C- Ly6G- I-A/I-E+ F4/80+ cells among CD45+ live cells. DC were identified as

CD11b+ Ly6C- Ly6G- I-A/I-E+ F4/80- CD11c+ cells among CD45+ live cells. CD4T were identified as CD11b- CD19- CD3+ CD4+ CD8- cells among CD45+ live cells. CD8T were identified as CD11b- CD19- CD3+ CD4- CD8+ cells among CD45+ live cells. B were identified as CD11b- CD3- CD19+ I-A/I-E+ cells among CD45+ live cells. Each cytokine-positive gate was determined based on a Fluorescence Minus One (FMO) control.

☒ Tick this box to confirm that a figure exemplifying the gating strategy is provided in the Supplementary Information.
