# Supplementary material for: Thymulin restrains age-associated myeloid inflammation and enhances cancer immunotherapy
Source: Nat Commun. 2026 Jul 21;17:6534. doi: 10.1038/s41467-026-75383-0 (PMC13389034; doi:10.1038/s41467-026-75383-0)
Supplement: Supplementary file 7 — Transparent Peer Review file [file 41467_2026_75383_MOESM7_ESM.pdf]

nature portfolio

Peer Review File

Thymulin restrains age-associated myeloid inflammation and enhances cancer immunotherapy

Corresponding Author: Dr Fumito Ito

This file contains all reviewer reports in order by version, followed by all author rebuttals in order by version.

Version 0:

Reviewer comments:

Reviewer #1

(Remarks to the Author)
Despite the well-established observation of enhanced systemic and peripheral inflammation and inflammatory cytokines with age ("inflammaging") and its association with various diseases including cancer, the sources and drivers of inflammaging are not completely understood and so the authors are asking an important and relevant question. They performed a series of experiment (mostly flow cytometry based along with cytokine analysis) to broadly and very generally characterize inflammaging in c57Bl/6 mice with and without tumors. Parabiosis and bone marrow chimeras led them to the conclusion that exposure to a young systemic environment reverses some of the inflammaging hallmarks and attenuates tumor progression and that a soluble factor is not bone marrow derived. They identifyed this factor as thymus-derived thymulin, which they suggest suppresses inflammaging and attenuates tumor growth. The findings are interesting and relevant but some of the arguments do not consider alternative explanations without further experimentation and there are some weaknesses that could be addressed to strengthen the manuscript

1. The authors should consider revising their title to convey the specific findings, rather than a title that is broad and rather vague. For example, their Running Title is more informative and descriptive of the work that is presented.

2. Given that age-related factors and mechanisms of cancer progression vary significantly across cancer types, it would be helpful to include the cancer types in their abstract, rather than just stating "…these myeloid cells are found in the tumor". Please specify which cancer types/tumor models were studied.

3. Line 86. It is critical to specify which mouse strain(s) were used for these initial observational analyses of "increased inflammation". Mouse strains vary widely in their peripheral and tissue-resident immune profiles. While the flow cytometry profiles are convincing, these observations would benefit from some type of validation (literature or existing scRNAseq datasets on similarly young and aged mice), or, if those are not available, then a discussion of how mouse strains can vary widely and a rationalization of why they only focused on the Bl/6 model.

4. Fig. 1. Given the association the authors make between age-dependent peripheral and intratumoral inflammatory myeloid cells in the TNBC mouse model, and the elevated inflammatory myeloid cells in human PBMCs, it might strengthen their claims to know whether similar features of intratumoral myeloid cells are observed in human TNBC tumors. This could be done from published datasets of human breast cancer with age (Parsons, et al., Nature Aging, 2025).

5. Fig. 2. Although similar parabiosis experiments have been done, they could still learn something important. While the A-Y pairs show decreased tumor progression and improved survival in the aged mice, is the converse true? Do young parabionts experience accelerated tumor progression?

6. Fig. 3. It seems there could be other alternative explanations for the results of the bone marrow chimeras than solely concluding that the age-related inflammation is not of bone marrow origin. Can the authors show that there are no engraftment differences to explain why aged (or young) myeloid progenitors or myeloid cells would not engraft or transplant similarly? Or at the very least, is there any existing literature to this effect?

7. The data in Fig.5 and 6 are important and interesting and suggest thymulin suppressed aging-related inflammation. Is there any evidence to suggest thymulin levels are regulated or altered during pro-inflammatory states or that it plays a role in

resolving inflammation? Likewise, it may be important to show whether thymectomy in young mice can recapitulate the aging-related tumor progression, as the authors only showed the effects of gain-of-function.

8. Is there any evidence that thymulin levels decrease with age in humans or what thymulin status is in cancer patients?

9. It would be interesting to investigate, or report if it's known, whether Nude mice (athymic) have elevated peripheral inflammation and if they recapitulate some of the aging phenotype reported here.

Reviewer #2

(Remarks to the Author)
Kanemaru, H. et al. present a manuscript titled "A determinant of age-related myeloid cell-driven chronic inflammation and cancer progression" that demonstrates a higher frequency of myeloid cells producing the pro-inflammatory cytokines IL-1α, IL-1β, IL-6 and TNF-α occurs with aging and is associated with increased tumor growth. The authors further demonstrate that thymulin reduces these pro-inflammatory cytokines produced in myeloid cells and increased activated intratumoral T cells, delays tumor growth and enhances anti-PD-L1 response in aged mice. The authors presents extensive in vivo studies using two breast cancer models, parabiosis and bone marrow chimera studies, and human blood samples. While the work presented is important to the field, the manuscript would benefit from several major and minor revisions, which are provided for the authors' consideration to better support the conclusions.

Major:
• The current introduction and discussion primarily address chronic inflammation and cancer in a broad context. However, since all of the preclinical studies in this manuscript are conducted specifically in breast cancer models, these sections would be strengthened by incorporating a more focused discussion of pro-inflammatory cytokines in relation to breast cancer. Additionally, the findings derived from the two preclinical breast cancer models should be interpreted within this specific disease context, as they may not be readily generalizable to other cancer types.
• A brief explanation of how the ages of the mice used in this study (8–12 weeks for young mice and 65–75 weeks for aged mice) correspond to stages of human aging would help contextualize the findings in terms of human physiology. Additionally, the manuscript would benefit from grouping the human pro-inflammatory cytokine data into comparable age categories, similar to the mouse cohorts, to better highlight the parallel findings across species.
• Neutrophils are an important myeloid cell subset that has been implicated in chronic inflammation. It would be helpful for the authors to clarify why $CD11b^{+}Ly6G^{+}$ neutrophils were not included in the analysis.
• The authors attribute the increase in pro-inflammatory cytokines as IL-1α, IL-1β, IL-6, and/or TNF-α expression primarily to aging. However, tumor presence may also contribute to changes in these cytokines. The manuscript would be strengthened by including a comparison between healthy (non–tumor-bearing) and tumor-bearing mice, and discussing how tumor-induced inflammation may influence myeloid cell dysregulation in the context of aging
• In the absence of young-to-aged bone marrow chimera experiments, it is difficult to conclusively determine that non–bone marrow–derived soluble factors are solely responsible for driving the production of pro-inflammatory cytokines.
• Monocytes appear to be the myeloid cell subset exhibiting the highest expression of IL-1α, IL-1β, IL-6, and TNF-α; however, only macrophages were further examined for NF-κB signaling. It would be helpful for the authors to clarify the rationale for focusing specifically on macrophages rather than monocytes.
• The authors conclude on page 10, line 201 that "Taken together, these results suggest that thymulin…leading to reduction of pro-inflammatory cytokine expression in aged myeloid cells." However, the experiments presented in Figure 5 were conducted only in aged mice and human blood cells. Without including comparable analyses in young cells, it is difficult to determine whether the observed effects of thymulin are specific to aging or represent a more general impact on myeloid cytokine expression. Including data from young cohorts, or discussing this limitation, would strengthen the interpretation
• Page 9, line 194: As currently written, this statement may be misleading, as the data in Figure 5I indicate that thymulin downregulates NF-κB specifically in LPS-activated macrophages. Clarifying this context would improve accuracy.
• In the Results section titled "Thymulin controls tumor growth and renders tumors sensitive to anti–PD-L1 therapy in aged mice", the conclusions are based on experiments presented in Figure 6, which were performed exclusively in aged mice. Without corresponding thymulin treatment studies in young mice, it is difficult to determine whether the observed sensitization to anti–PD-L1 therapy is specific to aged mice or reflects a broader effect of thymulin across age groups. Including data from young cohorts, or discussing this limitation, would strengthen the interpretation. Additionally, it would be helpful for the authors to clarify whether IFN-γ–producing $CD4^{+}$ and $CD8^{+}$ T cells are increased in young mice, which could potentially contribute to the delayed tumor growth observed compared to aged mice.
• Minimal discussion on the translational relevance of thymulin.

Minor:
• Figure 1B legend – clarify that this is mouse CD11b+ blood cells.
• Page 5, line 94 – as written, it sounds like a syngeneic and an orthotopic breast cancer model was used, but both AT-3 and E0771 are syngeneic models and the methods states both cell lines were implanted orthotopically in the 4th mammary fat pad. Thus, this should be changed to "we utilized two syngeneic orthotopic breast cancer mouse models" for clarity.

Reviewer #3

(Remarks to the Author)
This study conducted in aged mice and correlated to human PBMC analyses demonstrates that myeloid cells with pro-inflammatory secretory signature increase in circulation with age, and that their recruitment to tumors is pro-tumorigenic. Shared circulation of blood from young mice, achieved through parabiosis, is able to mitigate age-associated myeloid activation within aged mice, suggesting a circulating restorative factor. Experiments in heterochronic bone marrow chimeras further suggest that this circulating factor comes from non-hematopoietic cells, to which they mine public datasets to determine that thymulin, secreted from the thymus, is the circulating factor that reduces myeloid activation. Finally, they demonstrate that addition of thymulin to checkpoint blockade regimen is able to improve tumor outcomes in an age-dependent manner by modulating myeloid signatures. The study is well executed and its results are attention grabbing, as it suggests that thymulin from the thymus is an important factor in maintaining immunologic youth.

1. In the heterochronic mixed bone marrow chimeras more supplemental data is requested to boost the significance of the findings. The congenic background of the young or aged host is unclear, presumably it is CD45.2. While ideally irradiation would cause the complete ablation of the host hematopoietic compartment, there is the potential for residual host immune cells to evade irradiation. If so, then the CD45.2+ fraction may represent both donor and host immune cells, which could increase the apparent inflammatory signature in the myeloid cells of Y+A->A recipients. Also requested is data showing the reconstitution of the immune compartment overall. What % of the myeloid compartment is composed of CD45.1+ versus CD45.2+ in the young versus aged recipients?

Other comments:
2. line 95 - it would be helpful to know if the frequencies of CD45+ immune cell infiltration is comparable between young and aged mouse tumors, the data is expressed as % of CD45+ or other immune cell subset.
3. line 167 - more description of how the IPA databaset exploration was conducted is needed, there are no references and also is absent from the Methods.

Reviewer #4

(Remarks to the Author)
The authors interrogate the cellular and systemic basis of inflammaging and its functional consequences for tumor progression in aged hosts. They demonstrate that aged mice accumulate circulating and intratumoral CD11b^{+} myeloid populations that produce elevated IL-1α, IL-1β, IL-6, and TNF-α, a phenotype mirrored in human peripheral blood with increasing age. In orthotopic breast cancer models, this myeloid cytokine bias associates with accelerated tumor growth and inferior survival in aged animals. Through heterochronic parabiosis and congenic tracking, the authors show that exposure of aged mice to a youthful systemic milieu attenuates cytokine production in aged-derived myeloid cells and delays tumor progression. Bone marrow chimera experiments further localize this regulatory effect to non–bone marrow-derived circulating factors, with host age dictating the inflammatory tone of hematopoietic cells. IPA analysis and prior genetic evidence implicating thymic involution led the authors to examine thymulin. Thymulin administration reduces pro-inflammatory cytokine production in aged murine myeloid cells and human PBMCs, mechanistically linked to suppression of NF-κB activation. In vivo, thymulin diminishes intratumoral myeloid cytokine expression, enhances T-cell effector function, delays tumor growth in aged mice, and restores sensitivity to anti–PD-L1 therapy.
Collectively, the data support a model in which thymic decline contributes to age-associated myeloid inflammation through loss of thymulin, thereby constraining anti-tumor immunity. Restoration of thymulin signaling partially reconditions the aged immune microenvironment and improves checkpoint inhibitor efficacy, suggesting a systemic, thymus-dependent axis that modulates tumor control in the context of aging.

Overall a well written manuscript describing an exciting collection of findings. My critiques are minimal. IF they were correct that the effect is non-bone marrow, then transplanting Y bone marrow into A hosts should recreate the high inflammatory phenotype. Furthermore, if the thymus is the true source of the effect, then thymectomy in a Y animal will also recapitulate the phenotype of aging.

Version 1:

Reviewer comments:

Reviewer #1

(Remarks to the Author)
The authors have done an excellent job to address the concerns regarding data robustness and interpretation, and have significantly strengthened the work. The only remaining suggestion is that, in the absence of thymectomy requested by 2 reviewers, they tone down the conclusion that bone marrow and other sources are not involved and that the sole effect is due to thymus. Changing this language would provide clarity for the research community. Otherwise, this work is excellent and the authors should be commended for their efforts.

Reviewer #2

(Remarks to the Author)

A well-written manuscript and the revisions address all previously raised comments. One minor comment regarding proofreading the manuscript for spelling and typographical errors, e.g., page 7, line 145, “peripheral” is misspelled.

Reviewer #3

(Remarks to the Author)
Thank you to the authors for thoughtful revision, I have no concerns.

Reviewer #4

(Remarks to the Author)
authors have addressed my critiques

Open Access This Peer Review File is licensed under a Creative Commons Attribution 4.0 International License, which permits use, sharing, adaptation, distribution and reproduction in any medium or format, as long as you give appropriate credit to the original author(s) and the source, provide a link to the Creative Commons license, and indicate if changes were made.
In cases where reviewers are anonymous, credit should be given to 'Anonymous Referee' and the source.
The images or other third party material in this Peer Review File are included in the article’s Creative Commons license, unless indicated otherwise in a credit line to the material. If material is not included in the article’s Creative Commons license and your intended use is not permitted by statutory regulation or exceeds the permitted use, you will need to obtain permission directly from the copyright holder.
To view a copy of this license, visit https://creativecommons.org/licenses/by/4.0/

RE: Revision of Manuscript [NCOMMS-25-96627-T]

Title: A determinant of age-related myeloid cell-driven chronic inflammation and cancer progression

New Title: Thymulin restrains age-associated myeloid inflammation and enhances cancer immunotherapy

Dear Reviewers,

We appreciate for your careful reading and thoughtful comments. Our major revisions are highlighted in yellow throughout the body of the manuscript. In response, we have substantially revised the manuscript and believe that these changes have significantly strengthened the study. In particular, we have incorporated extensive new experimental data, including 62 additional display items across multiple figures (**Figures 2b–h, 4b, 5i–k, 6c–h, 7c–g,** and **Supplemental Figures 3a-c, 4a-d, 5a,b, 7b, c, e, f, h, 8b-h, 9a-d, 10a-d, 11a-e, 12a-e**). We have also clarified key interpretations, expanded the Discussion, and refined the title to better reflect the central findings. The original comments are reproduced below accompanied by a point-by-point set of responses in blue text.

Response to Reviewer #1

The findings are interesting and relevant but some of the arguments do not consider alternative explanations without further experimentation and there are some weaknesses that could be addressed to strengthen the manuscript.

We thank the reviewer for the positive comments on our manuscript, and would like to address some of the arguments and weaknesses.

1. *The authors should consider revising their title to convey the specific findings, rather than a title that is broad and rather vague. For example, their Running Title is more informative and descriptive of the work that is presented.*

We thank the reviewer for this helpful suggestion. We agree that the original title was too broad and did not fully convey the specific findings of our study. We have therefore revised the title to more clearly reflect the role of thymulin in restraining age-associated chronic inflammation in myeloid cells and enhances cancer immunotherapy.

2. *Given that age-related factors and mechanisms of cancer progression vary significantly across cancer types, it would be helpful to include the cancer types in their abstract, rather than just stating "...these myeloid cells are found in the tumor". Please specify which cancer types/tumor models were studied.*

Thank you for this important comment. We agree that age-related mechanisms of cancer progression can differ across tumor types. In our study, we evaluated age-associated tumor progression and characterized tumor-infiltrating myeloid cells using AT-3 and E0771 syngeneic murine mammary carcinoma models. In the revised manuscript, we also included findings using B16 melanoma (**new Supplementary Fig. 3c**) and EMT6 mammary carcinoma (**new Supplementary Fig. 11e**) models as complementary models. Since the majority of the data came from the mouse models of breast cancer, we revised the Abstract (page 2, lines 27–30) as follows "These cells are further enriched within the breast tumor microenvironment in aged mice and are associated with accelerated tumor progression."

3. *Line 86. It is critical to specify which mouse strain(s) were used for these initial observational analyses of "increased inflammation". Mouse strains vary widely in their peripheral and tissue-resident immune profiles. While the flow cytometry profiles are convincing, these observations would benefit from some type of validation (literature or existing scRNAseq datasets on similarly young and aged mice), or, if those are not available, then a discussion of how mouse strains can vary widely and a rationalization of why they only focused on the Bl/6 model.*

We thank the reviewer for this important comment. The majority of observational analyses were performed using C57BL/6 mice, which is now clearly stated in the revised manuscript. To address potential strain-specific effects, we analyzed aged and young BALB/c mice and observed a similar age-associated increase in myeloid cell inflammatory activation, accelerated tumor progression and efficacy of thymulin (**new Supplementary Fig. 11a-e**). Accordingly, we mentioned that our findings should be interpreted within this disease context in the discussion. These additions have been incorporated into the revised manuscript. We have now addressed these points in the Results (page 5, line 96-97; page 13, lines 267–268) and the Discussion (page 18, lines 395-398) section of the revised manuscript.

4. *Fig. 1. Given the association the authors make between age-dependent peripheral and intratumoral inflammatory myeloid cells in the TNBC mouse model, and the elevated inflammatory myeloid cells in human PBMCs, it might strengthen their claims to know whether similar features of intratumoral myeloid cells are observed in human TNBC tumors. This could be done from published datasets of human breast cancer with age (Parsons, et al., Nature Aging, 2025). https://www.nature.com/articles/s43587-025-00984-1*

We thank the reviewer for this insightful suggestion. In response, we analyzed a published human breast cancer dataset with age annotation (Parsons et al., *Nature Aging*, 2025; GEO accession: GSE176078) to assess whether age-associated features of intratumoral myeloid cells observed in our mouse model are also present in human TNBC tumors.
Using this dataset, we examined transcriptional profiles of tumor-infiltrating myeloid cells across age groups. Consistent with our findings in mice, myeloid cells from aged patients exhibited enrichment of pathways associated with inflammatory responses, including IL-1, IL-6, and TNF signaling.
These results support the presence of age-dependent inflammatory reprogramming of myeloid cells not only in the peripheral compartment but also within the human tumor microenvironment (**new Fig. 2c-h**). We have now included these points in the Results (pages 6–7, lines 118–144) and the Methods (pages 26–27, lines 585–608) section of the revised manuscript.

5. *Fig. 2. Although similar parabiosis experiments have been done, they could still learn something important. While the A-Y pairs show decreased tumor progression and improved survival in the aged mice, is the converse true? Do young parabionts experience accelerated tumor progression?*

We thank the reviewer for this important comment. To directly address whether the converse is true, we established additional parabiosis cohorts consisting of Young-Young (Y-Y), Aged-Young (A-Y), and Aged-Aged (A-A) pairs, and implanted E0771 tumors into the opposite parabiont. Importantly, we found that increased progression of tumors in young parabionts and worse survival in A-Y pairs compared with Y-Y pairs (**new Supplementary Fig. 7g, h**). Thus, the converse is indeed observed, indicating that exposure to an aged systemic environment promotes tumor growth even in young hosts. We have now included these points in the Results section (page 8, lines 158– 160) of the revised manuscript.

6. *Fig. 3. It seems there could be other alternative explanations for the results of the bone marrow chimeras than solely concluding that the age-related inflammation is not of bone marrow origin. Can the authors show that there are no engraftment differences to explain why aged (or young) myeloid progenitors or myeloid cells would not engraft or transplant similarly? Or at the very least, is there any existing literature to this effect?*

We thank the reviewer for this important comment. To address the possibility that differences in engraftment efficiency could account for the observed results, we evaluated donor chimerism using CD45.1/CD45.2 congenic markers. In all bone marrow chimeric mice, donor-derived hematopoiesis was very efficient: Young → Young: 99.3%, Aged → Young: 96.5%, and Aged → Aged: 99.6%. These data indicate that both young and aged bone marrow engraft effectively, and therefore the observed age-related inflammatory phenotypes are unlikely to be explained by differences in engraftment efficiency. We have now included these data in **new Fig. 4b**.
Importantly, donor chimerism within the CD11b+ myeloid compartment was similarly high across groups. Specifically, CD11b+ cells were predominantly CD45.1+ in Young → Young mice (98.3%) and predominantly CD45.2+ in Aged → Young (97.5%) and Aged → Aged (99.6%) mice, with minimal contribution from the alternate congenic marker in each case. These results demonstrate comparable myeloid reconstitution between young and aged donor bone marrow. The corresponding data have been added to **new Fig. 4b**. We have now addressed these points in the Results section (page 9, lines 180–181) of the revised manuscript.

7. *The data in Fig.5 and 6 are important and interesting and suggest thymulin suppressed aging-related inflammation. Is there any evidence to suggest thymulin levels are regulated or altered during pro-inflammatory states or that it plays a role in resolving inflammation? Likewise, it may be important to show whether thymectomy in young mice can recapitulate the aging-related tumor progression, as the authors only showed the effects of gain-of-function.*

We thank the reviewer for this important question. Although direct studies examining dynamic regulation of thymulin levels during acute inflammatory responses are limited, available evidence indicates that thymulin levels are altered under conditions of immune dysregulation. Circulating thymulin levels have been reported to be reduced in pathological inflammatory states, including autoimmune diseases (Safieh-Garabedian et al., *Journal of autoimmunity*, 1992), suggesting that thymulin deficiency may be associated with chronic inflammatory conditions. In parallel, a substantial body of experimental work supports a functional role of thymulin in regulating inflammatory responses. Thymulin has been shown to suppress pro-inflammatory cytokine

production (e.g., TNF-α, IL-1β, IL-6) and inhibit NF-κB signaling pathways (Safieh-Garabedian et al., *Int Arch Allergy Immunol,* 1993; Lunin et al., *Immunological investigations,* 2008; Novoselova et al., *PLoS One,* 2018; Lunin et al., *Arch Biochem Biophys,* 2023), consistent with a role in limiting or resolving inflammation.

Together, these findings support an immunomodulatory function of thymulin in inflammatory settings, although further studies will be required to define how thymulin levels are dynamically regulated during inflammatory responses in vivo, particularly in humans. We have now included these points in the Discussion (page 17-18, lines 378–380; page 18, lines 380 - 383) section of the revised manuscript.

We thank the reviewer for this important suggestion and agree that loss-of-function approaches would provide valuable complementary evidence. We expect that thymectomy in young mice would recapitulate key features of aging-associated inflammation and tumor progression. Indeed, recent human studies support a link between thymic function and systemic inflammation and cancer outcomes. Thymectomy in adults has been associated with increased systemic inflammation and higher risks of cancer and mortality (Kooshesh et al., *N Engl J Med*, 2023), and additional studies demonstrate that thymic function correlates with chronic inflammation (Bernatz et al., *Nature*, 2026 PMID: 41851466), tumor progression, and immunotherapy responses (Bernatz et al., *Nature*, 2026 PMID: 41851467).

However, thymectomy represents a global perturbation of thymic function and does not specifically model thymulin loss-of-function. The thymus produces multiple factors, including thymosin α1, thymosin β4, thymulin, and thymopoietin, and is also essential for T cell development. As a result, thymectomy induces broad immunological changes, making it difficult to attribute any observed phenotype specifically to thymulin.

Given that the primary goal of this study is to define the role of thymulin in regulating age-associated myeloid inflammation and tumor progression, we focused on gain-of-function approaches that allow more direct mechanistic interpretation.

We have therefore clarified this point in the revised Discussion (page 16-17, lines 353–359) and expanded our discussion of thymic regulation of age-associated inflammation.

8. *Is there any evidence that thymulin levels decrease with age in humans or what thymulin status is in cancer patients?*

We thank the reviewer for this important question. Evidence suggests that circulating thymulin levels decline with age in humans. A study analyzing plasma thymulin levels across individuals from infancy to old age reported that thymulin levels peak during childhood and progressively decrease after adolescence (Bach et al., *Ann N Y Acad Sci.*, 1975; Consolini et al., *Clin. Exp. Immunol*., 2000).

In addition, reduced thymulin activity has been reported in cancer patients. Consolini et al. demonstrated that young cancer patients exhibit significantly lower circulating thymic hormone (FTS/thymulin) activity compared with age-matched healthy controls, with undetectable levels in a substantial proportion of patients (Consolini et al., *Clin. Exp. Immunol*., 1986). We have now included these points in the Discussion section (pages 17-18, lines 377–380) of the revised manuscript.

9. *It would be interesting to investigate, or report if it's known, whether Nude mice (athymic) have elevated peripheral inflammation and if they recapitulate some of the aging phenotype reported here.*

We thank the reviewer for this insightful suggestion. We agree that the relationship between thymic deficiency and systemic inflammation is an important question. In the revised manuscript, we have added a statement to address the potential relationship between thymic deficiency and systemic inflammation.
Nude (*Foxn1*-null) mice lack a functional thymus and fail to generate mature T cells; however, available evidence regarding their peripheral inflammatory status, particularly in the context of aging, remains limited and somewhat inconclusive. However, age-associated decline in *Foxn1* expression has been linked to thymic dysfunction and increased levels of systemic inflammatory cytokines, including IL-6 and IL-1β (Oh et al., *JCI Insight* 2020), supporting a role for thymic integrity in regulating inflammatory tone. We have now addressed these points in the Discussion (page 16, lines 350–353) section of the revised manuscript.

Response to Reviewer #2

We thank the reviewer #2 for the comments on our manuscript.

1. The current introduction and discussion primarily address chronic inflammation and cancer in a broad context. However, since all of the preclinical studies in this manuscript are conducted specifically in breast cancer models, these sections would be strengthened by incorporating a more focused discussion of pro-inflammatory cytokines in relation to breast cancer. Additionally, the findings derived from the two preclinical breast cancer models should be interpreted within this specific disease context, as they may not be readily generalizable to other cancer types.

We thank the reviewer for this important comment and agree that a more focused discussion of pro-inflammatory cytokines in the context of breast cancer would strengthen the manuscript. Accordingly, we have revised both the Introduction and Discussion to better contextualize our findings within breast cancer biology and the inflammatory tumor microenvironment.
To further address concerns regarding disease specificity, we have incorporated additional data from complementary preclinical models, including the EMT6 breast cancer model and the B16 melanoma model (**new Supplementary Figs. 3 and 11**). These analyses demonstrate that key features of the phenotype, including age-associated inflammation and responsiveness to thymulin, are observed across multiple tumor models, suggesting that the underlying mechanisms may not be restricted to breast cancer.
At the same time, we acknowledge that the primary experimental framework of this study is based on breast cancer models (AT-3 and E0771). We have therefore added a statement in the Discussion explicitly noting that our findings should be interpreted within this disease context and that further studies will be required to determine their generalizability to other cancer types. We have now included these points in the Introduction (page 3, lines 50 – 52), Results (page 5, lines 98–99; page 13, lines 267–268), and Discussion (page 14, lines 306 - 308; page 18, lines 390–398) sections of the revised manuscript.

2. A brief explanation of how the ages of the mice used in this study (8–12 weeks for young mice and 65–75 weeks for aged mice) correspond to stages of human aging would help contextualize the findings in terms of human physiology. Additionally, the manuscript would benefit from grouping the human pro-inflammatory cytokine data into comparable age categories, similar to the mouse cohorts, to better highlight the parallel findings across species.

We thank the reviewer for this insightful suggestion. We have revised the manuscript to clarify the relationship between mouse and human age and to improve cross-species comparability.
First, we have added a brief explanation in the Methods section to contextualize mouse ages in relation to human aging. Based on established lifespan comparisons in C57BL/6 mice (Dutta & Sengupta, 2016), mice at 8–12 weeks of age are considered young adults, corresponding approximately to humans in early adulthood (approximately 20–30 years). In contrast, mice at 65–75 weeks are considered aged and correspond approximately to humans in late middle to early elderly stages (approximately 50–70 years), depending on the physiological parameter assessed. These references have now been cited in the Methods section of revised manuscript (page 19, lines 408–415).

Second, following the reviewer's recommendation, we reanalyzed the human pro-inflammatory cytokine data using age categories designed to parallel the murine cohorts. Based on cross-species age equivalency models and consistent with the World Health Organization definition (https://www.who.int/news-room/fact-sheets/detail/ageing-and-health) of older adults (≥60 years), we defined the human aged group as individuals aged 60 years and older. Importantly, pro-inflammatory cytokine levels showed significant elevation beginning at this threshold, supporting its biological relevance. This age stratification allowed clearer visualization of age-associated trends and improved cross-species comparison. The revised analyses are presented in **new Fig. 2b** and described in the Results section (page 6, lines 116–117) of the revised manuscript.

3. *Neutrophils are an important myeloid cell subset that has been implicated in chronic inflammation. It would be helpful for the authors to clarify why CD11b+Ly6G+ neutrophils were not included in the analysis.*

We thank the reviewer for this important comment. In our study, granulocytes were defined as CD11b+Ly6C+Ly6G+ cells, corresponding to granulocytes/neutrophils. This population was included in our analyses and exhibited comparable age-associated increases in cytokine production (Supplementary Figs. 2, 5c, d, and **new Supplementary Figs. 3b, 10d**). However, inflammatory signatures were more prominently enriched in monocyte/macrophage populations, consistent with observations in human breast tumors (ref. 24), which prompted us to focus subsequent mechanistic analyses on these subsets. We have clarified this in the Discussion (page 14, lines 305–308) of the revised manuscript.

4. *The authors attribute the increase in pro-inflammatory cytokines as IL-1α, IL-1β, IL-6, and/or TNF-α expression primarily to aging. However, tumor presence may also contribute to changes in these cytokines. The manuscript would be strengthened by including a comparison between healthy (non–tumor-bearing) and tumor-bearing mice, and discussing how tumor-induced inflammation may influence myeloid cell dysregulation in the context of aging.*

We thank the reviewer for this insightful comment and agree that tumor burden may contribute to systemic inflammatory cytokine levels in addition to aging. To address this, we have performed additional analyses comparing non–tumor-bearing and tumor-bearing mice at 2 weeks after tumor implantation in both young and aged cohorts using two tumor models (AT-3 and E0771) (**new Supplementary Fig. 4a–d**).
We observed a modest increase in circulating pro-inflammatory myeloid cells in tumor-bearing mice in both age groups, indicating that tumor presence contributes to expand inflammatory myeloid cells. However, the magnitude of this effect was substantially smaller than the difference observed between young and aged mice, demonstrating that aging is the predominant driver of the pro-inflammatory phenotype. Furthermore, age-associated increases in cytokine-producing myeloid

cells were consistently observed across tumor models and in non–tumor-bearing settings, and were reproducible across mouse strains (C57BL/6 and BALB/c) and in both male and female mice (Fig. 1a, **new Supplementary Figs. 3a, 11c**).
Together, these findings support a model in which tumor-induced inflammation acts as a context-dependent modulatory factor, whereas aging establishes a baseline pro-inflammatory state that shapes myeloid cell function. These results have been incorporated into the Results (page 5, lines 99–104; page 5, 93-95; page 13, 267–268) and the Discussion section (pages 7–8, lines 313–324) of the revised manuscript.

5. *In the absence of young-to-aged bone marrow chimera experiments, it is difficult to conclusively determine that non–bone marrow–derived soluble factors are solely responsible for driving the production of pro-inflammatory cytokines.*

We thank the reviewer for this insightful comment and agree that our original wording may have overstated the contribution of non–bone marrow–derived factors. Our intention was not to imply that these factors are solely responsible for pro-inflammatory cytokine production, but rather that the recipient environment plays a major role.
To directly address this point, we performed additional young-to-aged (Y→A) bone marrow chimera experiments. In this setting, both circulating and intratumoral young bone marrow–derived myeloid cells exhibited increased pro-inflammatory cytokine production in Y→A chimeras compared with Y→Y controls (**new Supplementary Fig. 8a-h**). These results demonstrate that the aged host environment is sufficient to promote inflammatory activation of myeloid cells, even when the donor bone marrow is young.
Together, these data support a model in which non–bone marrow–derived factors play a dominant role in shaping pro-inflammatory cytokine production in aged myeloid cells, while not excluding a contributory role for bone marrow–derived factors.
New data and clarification has been added to the Results (page 9, lines 191–196) and the Discussion section (page 15, lines 331–348) of the revised manuscript.

6. *Monocytes appear to be the myeloid cell subset exhibiting the highest expression of IL-1α, IL-1β, IL-6, and TNF-α; however, only macrophages were further examined for NF-κB signaling. It would be helpful for the authors to clarify the rationale for focusing specifically on macrophages rather than monocytes.*

We thank the reviewer for this important point. In the E0771 tumor model, monocytes exhibited the highest expression of inflammatory cytokines, whereas in the AT-3 model, macrophages represented the predominant inflammatory myeloid population (**new Fig. 7d, e,** Supplementary Figs. 5c, 5d, and **new Supplementary Figs. 5b, 7b, 7e, 8f**), indicating that the identity of the most pro-inflammatory subset is context-dependent and varies across tumor models.
For mechanistic studies, we focused on macrophages because tumor-associated macrophages represent a stable and functionally mature myeloid population within the tumor microenvironment and are well-established mediators of NF-κB–dependent inflammatory signaling. In contrast, monocytes are more transient and undergo differentiation upon tumor infiltration. In addition, to investigate NF-κB signaling under controlled and reproducible conditions, we utilized M-CSF–derived bone marrow–derived macrophages (BMDMs) and RAW264.7 cells, which are widely used and experimentally tractable systems for mechanistic studies.
Importantly, age-associated increases in pro-inflammatory cytokine production were observed across both monocyte and macrophage populations, supporting the conclusion that inflammaging broadly affects the myeloid lineage rather than a single subset. This clarification has been added to

the Results (page 6, lines 107-108) and the Discussion section (page 14, lines 297–308; page 15, lines 313–320) of the revised manuscript.

7. The authors conclude on page 10, line 201 that "Taken together, these results suggest that thymulin...leading to reduction of pro-inflammatory cytokine expression in aged myeloid cells." However, the experiments presented in Figure 5 were conducted only in aged mice and human blood cells. Without including comparable analyses in young cells, it is difficult to determine whether the observed effects of thymulin are specific to aging or represent a more general impact on myeloid cytokine expression. Including data from young cohorts, or discussing this limitation, would strengthen the interpretation.

We thank the reviewer for this important comment. We agree that the original version of Figure 5, which included only aged mice and human blood cells, did not allow us to determine whether the effects of thymulin were specific to aging.
To address this concern, we have now performed additional experiments using multiple young cohorts, including (i) in vivo blood samples from young mice (Blood Baseline), (ii) PBMCs from young human donors, and (iii) bone marrow–derived macrophages (BMDMs) from young mice. Across all of these young models, thymulin treatment did not produce a significant change in pro-inflammatory cytokine expression. In contrast, as shown in the original data, thymulin markedly reduced cytokine expression in aged myeloid cells. These results indicate that the effects of thymulin are not general but are instead specific to aged myeloid cells. These new data have been incorporated into the revised Figure 6 (see **new Fig. 6c-h**), and the Results and Discussion sections have been updated accordingly. We have also clarified this point in the text to emphasize the age-dependent effect of thymulin. This clarification has been added to the Results (page 12 lines 245–251) and the Discussion section (page 17, lines 370–375) of the revised manuscript.

8. Page 9, line 194: As currently written, this statement may be misleading, as the data in Figure 5I indicate that thymulin downregulates NF-κB specifically in LPS-activated macrophages. Clarifying this context would improve accuracy.

We thank the reviewer for this helpful comment. We agree that the original wording could be misleading, as the effect of thymulin on NF-κB activity was observed specifically in LPS-activated macrophages. We have revised the sentence to clarify this context. The text now reads: "Thymulin treatment reduced NF-κB p65 DNA-binding activity in nuclear extracts from LPS-activated bone marrow-derived macrophages of aged mice" (Page 12, Lines 253–255).

9. In the Results section titled "Thymulin controls tumor growth and renders tumors sensitive to anti–PD-L1 therapy in aged mice", the conclusions are based on experiments presented in Figure 6, which were performed exclusively in aged mice. Without corresponding thymulin treatment studies in young mice, it is difficult to determine whether the observed sensitization to anti–PD-L1 therapy is specific to aged mice or reflects a broader effect of thymulin across age groups. Including data from young cohorts, or discussing this limitation, would strengthen the interpretation. Additionally, it would be helpful for the authors to clarify whether IFN-γ–producing CD4+ and CD8+ T cells are increased in young mice, which could potentially contribute to the delayed tumor growth observed compared to aged mice.

We thank the reviewer for this important comment. As suggested, we have now included additional experiments in young mice to directly assess whether the effects of thymulin are age-dependent. In contrast to the findings in aged mice, thymulin treatment in young mice did not result in a significant antitumor effect, nor did it enhance the efficacy of anti–PD-L1 therapy (**new**

Supplementary Fig. 12e). These results indicate that the tumor-sensitizing and immunomodulatory effects of thymulin are age-dependent and preferentially observed in aged hosts.

Regarding the reviewer's question on IFN-γ–producing T cells, we analyzed CD4+ and CD8+ T cell responses in young mice and found no significant increase in IFN-γ–producing populations following thymulin treatment (**new Supplementary Fig. 12d**). This suggests that the enhanced antitumor immunity observed in aged mice is not recapitulated in young mice and may reflect age-associated immune modulation.

These findings suggest that thymulin preferentially enhances antitumor immunity in aged hosts, potentially by compensating for age-associated immune dysfunction, whereas its impact is limited in young mice where the thymulin level is maintained and stable (Bach et al., *Ann N Y Acad Sci.*, 1975).

We have incorporated these new data and clarified this point in the Results section (pages 13, lines 271–273; pages 13, lines 275–278)) and the Discussion (page 17, lines 370–375) of the revised manuscript.

10. *Minimal discussion on the translational relevance of thymulin.*

We thank the reviewer for this important comment. In the revised manuscript, we have expanded the Discussion to better address the translational relevance of thymulin. Specifically, we now highlight its potential to restore immune competence in aged individuals, particularly in the context of reduced responsiveness to immunotherapy. In addition, we discuss the importance of using aged murine models to more accurately reflect age-associated immune remodeling and improve the translational value of preclinical studies. These additions are included on page 17, lines 363–366 and 373–375.

11. *(Minor) Figure 1B legend – clarify that this is mouse CD11b+ blood cells.*

Thank you for your helpful comment. As you correctly pointed out, these are mouse $CD11b^+$ blood cells (Fig. 1b). We have revised the figure legend accordingly.

12. *Page 5, line 94 – as written, it sounds like a syngeneic and an orthotopic breast cancer model was used, but both AT-3 and E0771 are syngeneic models and the methods states both cell lines were implanted orthotopically in the 4th mammary fat pad. Thus, this should be changed to "we utilized two syngeneic orthotopic breast cancer mouse models" for clarity.*

Thank you for your helpful comment. We agree that the original wording may have been misleading. We have revised the sentence to "we utilized two syngeneic orthotopic breast cancer mouse models," as suggested, to improve clarity in the revised manuscript (page 5, lines 96–97).

Response to Reviewer #3

"The study is well executed and its results are attention grabbing, as it suggests that thymulin from the thymus is an important factor in maintaining immunologic youth."

We thank the reviewer for the positive comments on our manuscript.

1. In the heterochronic mixed bone marrow chimeras more supplemental data is requested to boost the significance of the findings. The congenic background of the young or aged host is unclear, presumably it is CD45.2. While ideally irradiation would cause the complete ablation of

the host hematopoietic compartment, there is the potential for residual host immune cells to evade irradiation. If so, then the CD45.2+ fraction may represent both donor and host immune cells, which could increase the apparent inflammatory signature in the myeloid cells of Y+A->A recipients. Also requested is data showing the reconstitution of the immune compartment overall. What % of the myeloid compartment is composed of CD45.1+ versus CD45.2+ in the young versus aged recipients?.

We thank the reviewer for this important and insightful comment. We have clarified the congenic backgrounds in the revised manuscript: young donor mice are CD45.1, whereas aged donor and recipient mice are CD45.2. To address the concern regarding incomplete ablation of the host hematopoietic compartment, we have included new data demonstrating the efficiency of bone marrow reconstitution. Donor engraftment exceeded 95% in all cohorts **(new Fig. 4b)**, indicating effective ablation of the host hematopoietic compartment and minimal contribution from residual host-derived cells. We have also included new analyses of donor chimerism in heterochronic mixed bone marrow chimeras. The proportions of CD45.1+ and CD45.2+ cells were comparable between experimental groups (**new Supplementary Fig. 9a**). Importantly, within the CD11b+ myeloid compartment, the relative contributions of CD45.1+ and CD45.2+ cells were similar across groups, indicating no bias in myeloid reconstitution and excluding the possibility that differential host cell contamination accounts for the observed inflammatory phenotypes. (**new Supplementary Fig. 9a)**.

In addition, we have provided new data characterizing the overall immune reconstitution in both blood and tumor tissues (**new Supplementary Fig. 9b, c**). These analyses revealed differences in immune composition between Y+A→Y and Y+A→A chimeras, with increased T cell abundance in Y+A→Y mice, potentially reflecting differences in thymic function.

To determine whether the observed effects on tumor progression and myeloid cell activation were independent of adaptive immunity, we generated heterochronic mixed bone marrow chimeras using Rag2-deficient mice as donors and recipients (**new Fig. 5h**). In the absence of T and B cells, the overall frequency of circulating CD11b+ myeloid cells remained comparable between Y+A→Y and Y+A→A chimeras (**new Supplementary Fig. 10a, b**). However, Y+A→Y mice continued to exhibit delayed tumor progression, improved survival, and reduced frequencies of cytokine-producing CD11b+ myeloid cells compared with Y+A→A mice (**new Fig. 5i-k** and **new Supplementary Fig. 10c, d**). Together, these results demonstrate that the observed differences in myeloid cell inflammatory activation are not attributable to incomplete irradiation or residual host-derived cells, but instead reflect a dominant role of host-derived factors in regulating myeloid cell function, independent of adaptive immunity. These data and clarifications have been incorporated into the Results (Results (page 9, lines 180–181; page 10, lines 204–206; page 10, lines 215–223) and the Discussion (page 16, lines 342–348) section of the revised manuscript.

2. line 95 - it would be helpful to know if the frequencies of CD45+ immune cell infiltration is comparable between young and aged mouse tumors, the data is expressed as % of CD45+ or other immune cell subset.

We thank the reviewer for this helpful suggestion. To determine whether overall immune cell infiltration differs between tumors from young and aged mice, we quantified total CD45+ immune cells normalized to tumor weight (CD45+ cells per mg tumor). As shown in the revised figure (**new Supplementary Fig. 5a**), the density of CD45+ immune cells per mg of tumor tissue was comparable between tumors from young and aged mice. We have now included these points in the Results section (page 5, lines 104–105) of the revised manuscript.

3. Lline 167 - more description of how the IPA databaset exploration was conducted is needed, there are no references and also is absent from the Methods

We thank the reviewer for this important comment. We agree that the description of the IPA database exploration was insufficient in the original manuscript.
In response, we have added a detailed description of the candidate selection process using the QIAGEN Ingenuity Pathway Analysis (IPA) database in the Methods section (page 26, lines 572–583). Briefly, 163 molecules associated with "Aging" in the "Diseases and Functions" category were initially identified, of which 35 were annotated as "Causal." These were further filtered based on relevance to longevity (9 molecules), and finally narrowed down to three factors (FOXO3, LMNA, and SIRT family members) based on reported increases in pro-inflammatory cytokines (IL-1, IL-6, and/or TNF-α) in knockout mouse models. The full list of initially identified molecules is now provided in **new Supplementary Data 3**.

Response to Reviewer #4

"Overall a well written manuscript describing an exciting collection of findings. My critiques are minimal."

We thank the reviewer for the positive comments on our manuscript.

1. IF they were correct that the effect is non-bone marrow, then transplanting Y bone marrow into A hosts should recreate the high inflammatory phenotype. Furthermore, if the thymus is the true source of the effect, then thymectomy in a Y animal will also recapitulate the phenotype of aging.

We thank the reviewer for this insightful comment.
To address the first point, we performed additional young-to-aged (Y→A) bone marrow chimera experiments. Consistent with the reviewer's prediction, transplantation of young bone marrow into aged hosts resulted in a significantly increased inflammatory phenotype compared with Y→Y controls (**new Supplementary Fig. 8a-h**). These findings demonstrate that the inflammatory state is not primarily determined by bone marrow–intrinsic properties, but rather by the host environment, supporting a dominant role for non–bone marrow–derived factors.
We have now included these points in the Results (page 9, lines 191–196) and the Discussion section (page 8, lines 335–337) of the revised manuscript.
With respect to the role of the thymus, we agree that thymic dysfunction represents a plausible component of this host environment. Indeed, recent human studies have shown that thymectomy in adults is associated with increased systemic inflammation and elevated risks of cancer and mortality (Kooshesh et al., *N Engl J Med*, 2023), and recent studies further demonstrate that thymic function correlates with chronic inflammation, tumor progression, and immunotherapy responses (Bernatz et al., [Thymic health consequences in adults] *Nature*. 2026 (PMID: 41851466); Bernatz et al., [Thymic health and immunotherapy outcomes in patients with cancer] *Nature*. 2026 (PMID: 41851467). These observations are consistent with our model and suggest that thymic insufficiency could contribute to the aging-associated inflammatory phenotype.
However, thymectomy represents a broad perturbation that affects multiple aspects of immune regulation, including loss of thymic hormones (e.g., thymosin α1, thymosin β4, thymulin, and thymopoietin) as well as impaired T cell development. As a result, while thymectomy may

recapitulate key features of age-associated inflammation, it would not provide a specific test of thymulin function. In contrast, our approach allows us to directly interrogate the role of thymulin in regulating myeloid cell inflammatory activation.

Taken together, these findings support a model in which thymic insufficiency contributes to inflammaging, while highlighting that targeted approaches are required to dissect the specific contribution of thymosin.

We have incorporated these clarifications into the Discussion (page 16, lines 350–357) of the revised manuscript.

RE: Revision of Manuscript [NCOMMS-25-96627-B]

Title: Thymulin restrains age-associated myeloid inflammation and enhances cancer immunotherapy

Dear Reviewers,

We appreciate for your careful reading and thoughtful comments. Our revisions are highlighted in yellow throughout the body of the manuscript. In response, we tone down the conclusion statements in the Result and Discussion sections. The original comments are reproduced below accompanied by a point-by-point set of responses in blue text.

Response to Reviewer #1

The authors have done an excellent job to address the concerns regarding data robustness and interpretation, and have significantly strengthened the work. The only remaining suggestion is that, in the absence of thymectomy requested by 2 reviewers, they tone down the conclusion that bone marrow and other sources are not involved and that the sole effect is due to thymus. Changing this language would provide clarity for the research community. Otherwise, this work is excellent and the authors should be commended for their efforts.

We thank the reviewer for the positive comments on our manuscript, and helpful suggestion. We changed "central role" to "important role" in the Result section in page 9, lines 183–186 and page 11, lines 220–222, and in the Discussion section in page 15, lines 328–330.

Response to Reviewer #2

A well-written manuscript and the revisions address all previously raised comments. One minor comment regarding proofreading the manuscript for spelling and typographical errors, e.g., page 7, line 145, "peripheral" is misspelled.

We thank the reviewer for the positive comments on our manuscript. We corrected the typographical errors.

INTERNAL
